# Supplementary material for: Disease-associated polyalanine expansion mutations impair UBA6-dependent ubiquitination
Source: EMBO J. 2024 Jan 2;43(2):5. doi: 10.1038/s44318-023-00018-9 (PMC10897158; doi:10.1038/s44318-023-00018-9)
Supplement: Supplementary file 1 — Appendix [file 44318_2023_18_MOESM1_ESM.pdf]

Appendix

Disease-associated polyalanine expansion mutations impair UBA6-dependent ubiquitination

(Amer-Sarsour et al.)

Table of Contents

Appendix Figure S1..2

Appendix Figure S2..3

Appendix Figure S3..4

Appendix Figure S4..5-6

Appendix Figure S5..7-8

A

Gene name	Ub function	NCBI Entrez Gene	Polyalanine location (protein residues)	Number of alanine residues
UBE2Z/USE1	E2	65264	47-52	6
BIRC6	E2/E3	57448	30-36, 1660-1666	7
MEX3C	E3	51320	177-184	8
RNF169	E3	254225	13-18	6
RN19B	E3	127544	92-97	6
SH3RF1	E3	57630	419-424	6
SH3RF3	E3	344558	13-18	6
TRIM24	E3	8805	10-15	6
UBR3	E3	130507	2-8	7
UBR5	E3	51366	1762-1768	7
ZFP91	E3	80829	59-67	9
ZNRF2	E3	223082	65-71	7
ZNF598	E3	90850	16-21	6

B

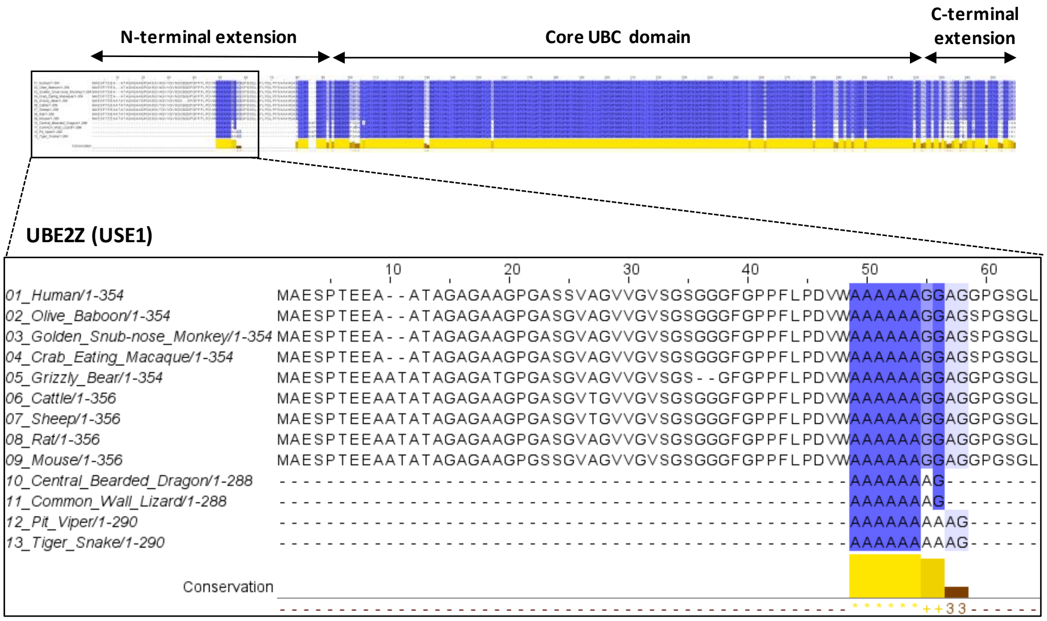

Appendix Figure S1. Analysis of alanine repeats in the ubiquitin system. A, Analysis of alanine repeats domains in the human ubiquitin cascades comprising E1, E2, and E3 enzymes. **B,** A multiple sequence alignment of USE1 homologs from different vertebrates. The alignment is colored according to sequence identity including the N-terminus containing the polyalanine stretch.

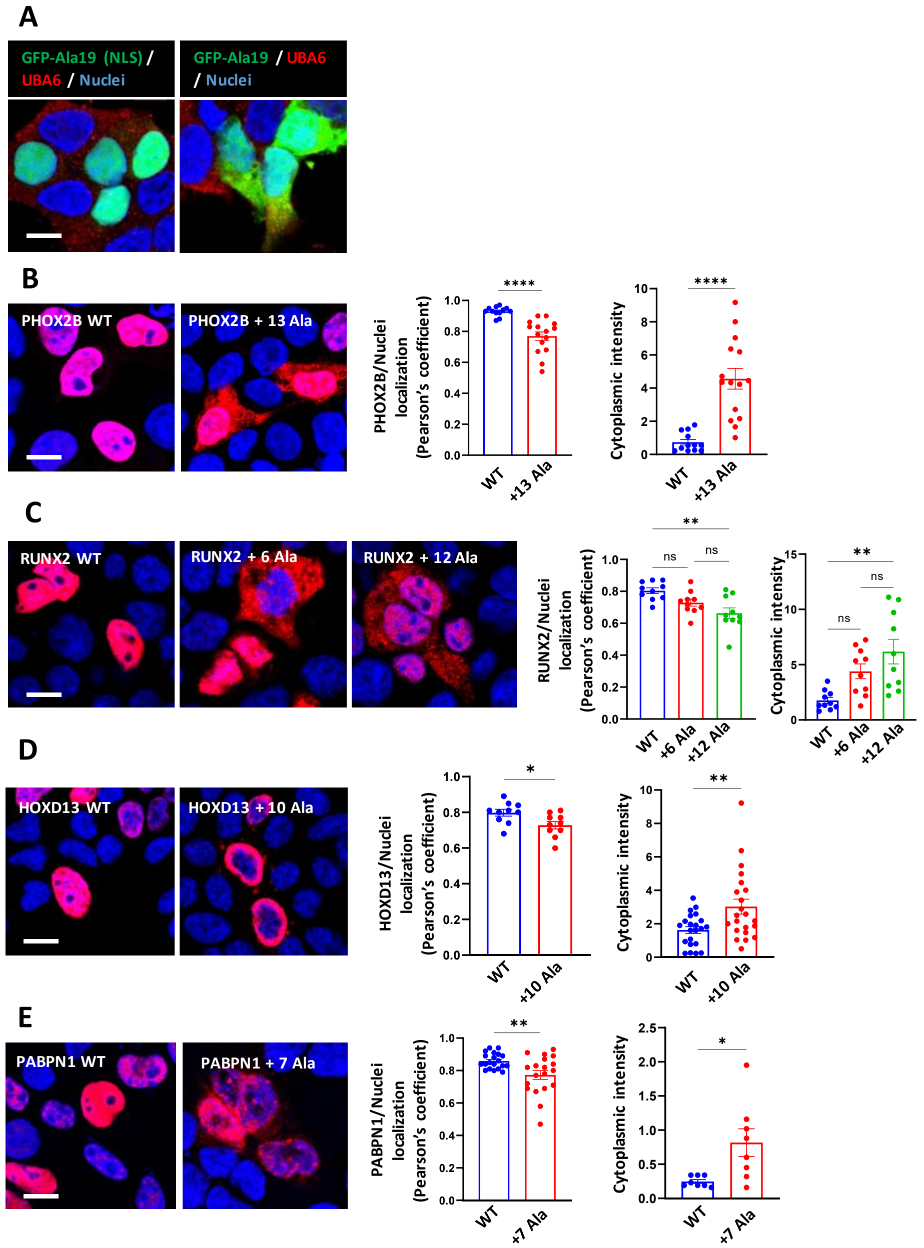

Appendix Figure S2. Polyalanine expansion mutations cause cytoplasmic mislocalization of different nuclear proteins. HEK293T cells were transfected with the indicated constructs, and were subjected to immunostaining. **A**, GFP-19Ala with nuclear localization sequence (NLS) or GFP-19Ala, labeled for endogenous UBA6. **B**, HA-PHOX2B WT and HA-PHOX2B +13Ala. **C**, HA-RUNX2 WT, HA-RUNX2 +6Ala, and HA-RUNX2+ 12Ala. **D**, HA-HOXD13 WT and HA-HOXD13 +10Ala. **E**, HA-PABPN1 WT and HA-PABPN1 +7Ala. Image scale bar 20 µm. Quantification of the association of HA-tagged proteins (labeled in red) with the nucleus (labeled in blue, Pearson's coefficient) is indicated as well as the cytoplasmic intensity. Results are the average values from cells in different imaged fields. Between 60-100 transfected cells were analyzed. Results are mean ± s.e.m. Unpaired 2-tailed t-test (**B, D, E)** and one-way ANOVA Tukey's test (**C**). ns non-significant, $*P < 0.05$, $**P < 0.01$, $****P < 0.0001$.

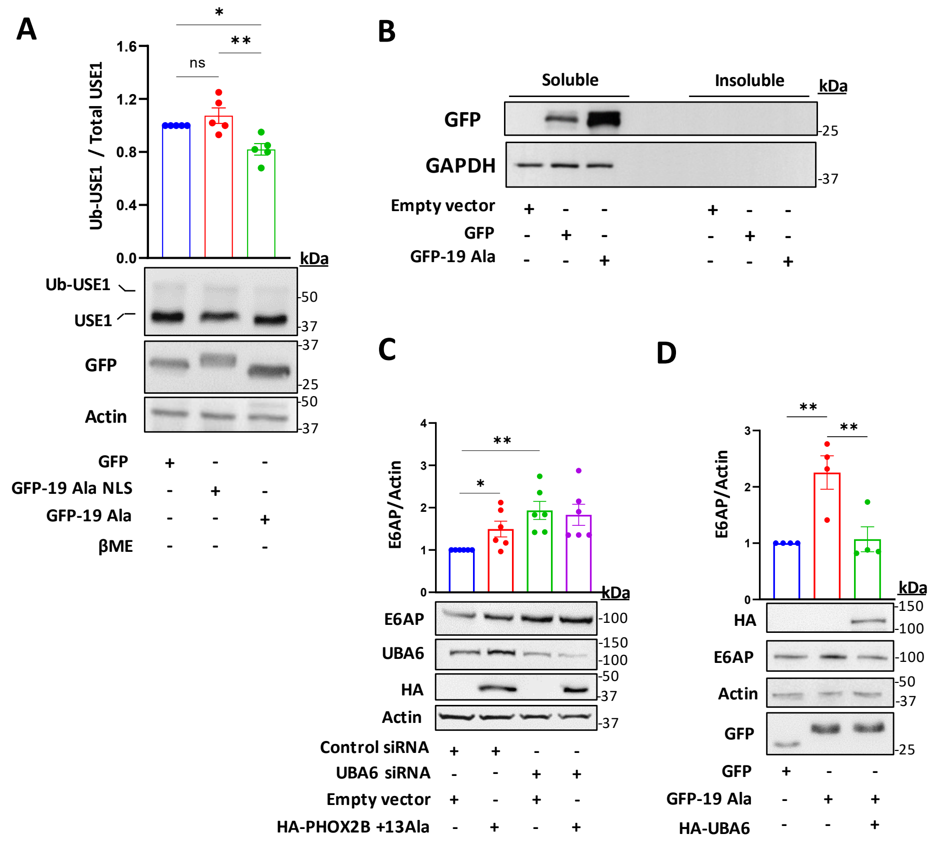

Appendix Figure S3. Soluble isolated polyalanine stretches regulate USE1 ubiquitin loading and E6AP levels. A, HEK293T cells were transfected with empty GFP, GFP-polyAla (19 Ala), and GFP-polyAla with a nuclear localization sequence (NLS). Endogenous USE1 ubiquitin loading was analyzed in βME-untreated cell lysates. **B**, HEK293T cells were transfected with empty GFP or GFP-polyAla. The cell lysates were analyzed for the soluble and sarkosyl-insoluble fractions of GFP-polyAla. **C**, Control and UBA6-depleted HEK293T cells were transfected with empty vector or mutant PHOX2B and analyzed for E6AP levels. **D,** Cells were transfected with empty GFP, or GFP-polyAla with or without HA-UBA6, and analyzed for E6AP levels. Results are mean ± s.e.m. (**A**) One-way ANOVA Tukey's test, n=5 (**B**) Unpaired 2-tailed t-test, n=6. (**D**) One-way ANOVA, Tukey's test, n=4. n.s not significant, *$P < 0.05$, **$P < 0.01$.

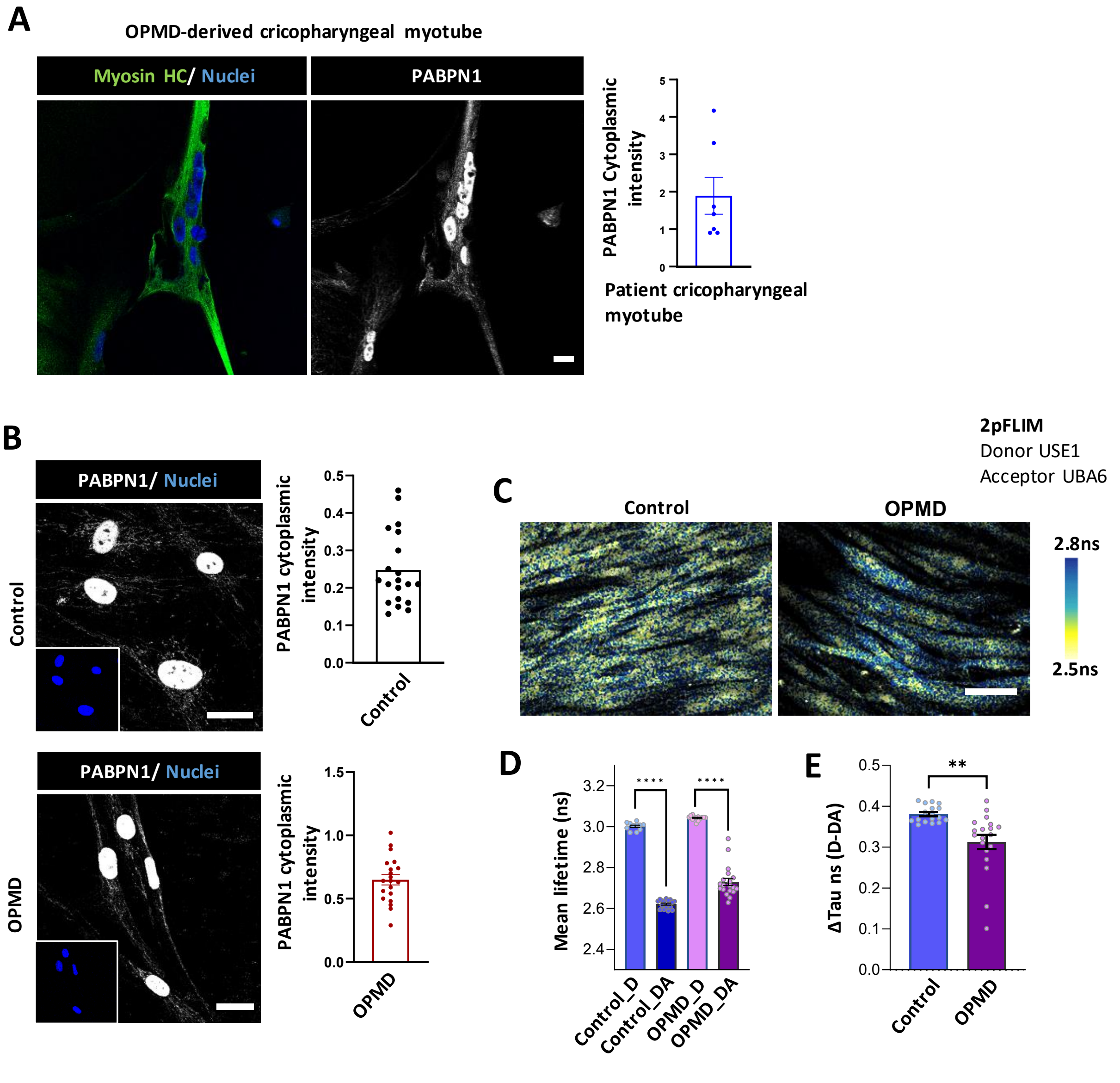

Appendix Figure S4. OPMD-patient derived cells exhibit cytoplasmic presence of PABPN1 and reduced association between UBA6 and USE1. A, OPMD patient-derived primary cricopharyngeal myotubes were stained for PABPN1, nuclei and myosin heavy chain (HC). Quantification of PABPN1 cytoplasmic intensity is presented as mean ± s.e.m (n=11 myotubes). **B**, Control and OPMD patient-derived primary fibroblasts were stained for PABPN1 and nuclei. The quantification of PABPN1 cytoplasmic intensity in different image fields is presented as mean ± s.e.m (Control n=538 cells, OPMD n=108 cells). **C**, Representative 2pFLIM pseudo colored images of control and OPMD fibroblasts, stained for USE1 and UBA6 using secondary antibodies as donor (Alexa 488) and acceptor (Alexa 555), respectively. Scale bar is 200µm. **D**, Comparison between donor only, and donor and acceptor lifetime in nano seconds (ns) for each group. For

control, donor only- 3.002±0.005 ns, donor/acceptor- 2.621±0.005 ns (mean ± s.e.m, n= 11 and 17 fields of view, respectively). For OPMD patient, donor only- 3.042±0.012 ns, donor/acceptor- 2.73±0.017 ns (mean ± s.e.m, n= 13 and 19 fields of view, respectively). **E**, Comparison of the difference in lifetime for each group, for the subtraction of donor only to donor and acceptor fluorescence lifetime. Control- 0.38±0.005, OPMD patient- 0.31±0.018 (mean ± s.e.m, n= 17 and 19 fields of view respectively). Unpaired 2-tailed t test. ** $P < 0.01$, ****$P < 0.0001$.

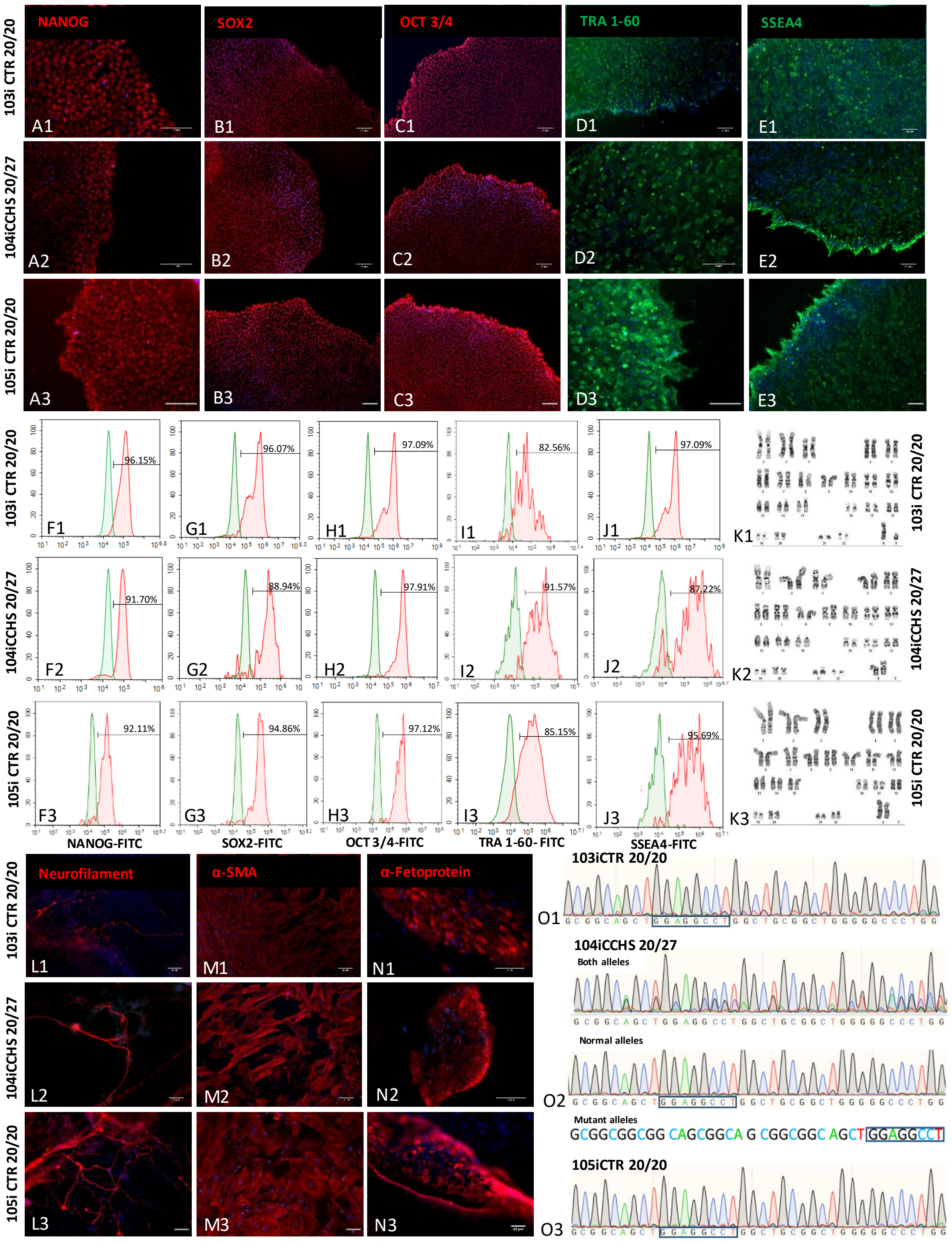
103i CTR 20/20
104iCCHS 20/27
105i CTR 20/20
NANOG
SOX2
OCT 3/4
TRA 1-60
SSEA4
A1
B1
C1
D1
E1
A2
B2
C2
D2
E2
A3
B3
C3
D3
E3
F1 96.15%
G1 96.07%
H1 97.09%
I1 82.56%
J1 97.09%
K1
F2 91.70%
G2 88.94%
H2 97.91%
I2 91.57%
J2 87.22%
K2
F3 92.11%
G3 94.86%
H3 97.12%
I3 85.15%
J3 95.69%
K3
NANOG-FITC
SOX2-FITC
OCT 3/4-FITC
TRA 1-60- FITC
SSEA4-FITC
Neurofilament
α-SMA
α-Fetoprotein
L1
M1
N1
L2
M2
N2
L3
M3
N3
103iCTR 20/20
O1
104iCCHS 20/27
Both alleles
Normal alleles
O2
Mutant alleles
GCGGCGGCGG CAGCGGCA G CGGCGGC AGCT GGAGGCCT
105iCTR 20/20
O3

Appendix Figure S5. Generation and characterization of CCHS patient and family relative-derived iPSCs. Skin punch biopsies were collected from a 2-year-old female patient with CCHS who harbors a heterozygous 27 polyalanine expansion in PHOX2B (104iCCHS 20/27), and from her healthy sister (105iCTR 20/20). A biopsy was also collected from the healthy father (103iCTR 20/20) of the 4-year-old male patients who harbors a heterozygous 25 polyalanine expansion in PHOX2B (102iCCHS 20/25). Patient-specific fibroblasts were electroporated with non-integrating reprogramming episomal plasmids. **A-E**, Immunocytochemistry for pluripotency markers (NANOG, SOX2, OCT3/4 TRA-1-60, SSEA4). **F-J**, Flow cytometry analysis for pluripotency markers (red-NANOG, SOX2, OCT3/4 TRA-1-60, SSEA4). **K**, G-banding karyotype. **L-N**, Embryoid bodies (EBs) were generated and allowed to spontaneously differentiate for 21 days. Differentiated EBs express the ectoderm marker heavy chain neurofilament, the mesoderm marker α- smooth muscle actin SMA, and the endoderm marker α-fetoprotein. **O**, Sequencing of the 3rd PHOX2B exon confirms the heterozygous +7 polyalanine expansion in the CCHS patient, but not in the healthy controls. Image scale bars 100 µm.
