# Supplementary figures and images for: Disease-associated polyalanine expansion mutations impair UBA6-dependent ubiquitination
Source: EMBO J. 2024 Jan 2;43(2):5. doi: 10.1038/s44318-023-00018-9 (PMC10897158; doi:10.1038/s44318-023-00018-9)

Figure 1

B

Ub-USE1  
FLAG  
USE1

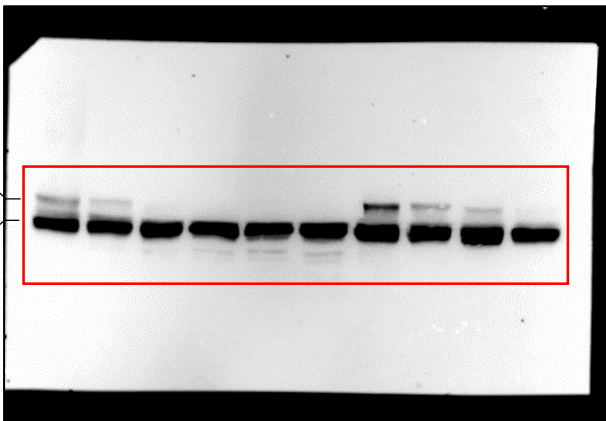

UBA6

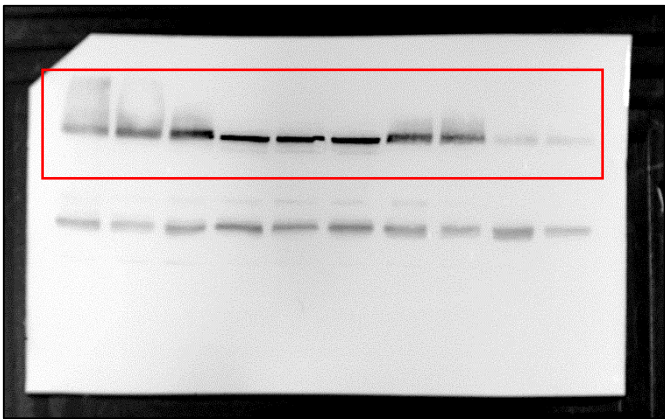

Actin

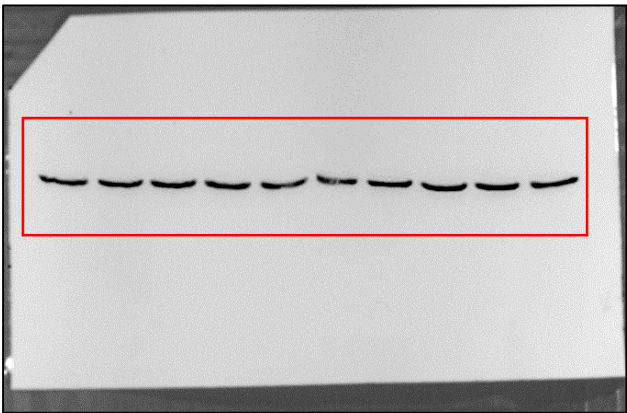

Supplement: Supplementary file 2 — Source Data Fig. 1 [file 44318_2023_18_MOESM2_ESM.zip › Figure 1/Fig 1B/Blot Fig 1B.pdf]

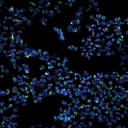

Supplement: Supplementary file 2 — Source Data Fig. 1 [file 44318_2023_18_MOESM2_ESM.zip › Figure 1/Fig 1D/Image 1D KO.tif]

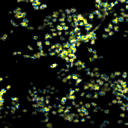

Supplement: Supplementary file 2 — Source Data Fig. 1 [file 44318_2023_18_MOESM2_ESM.zip › Figure 1/Fig 1D/Image 1D WT.tif]

Figure 1

E

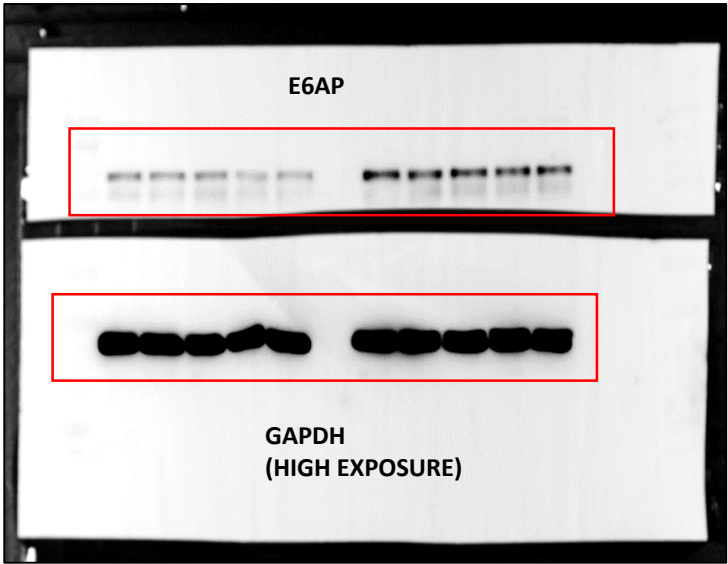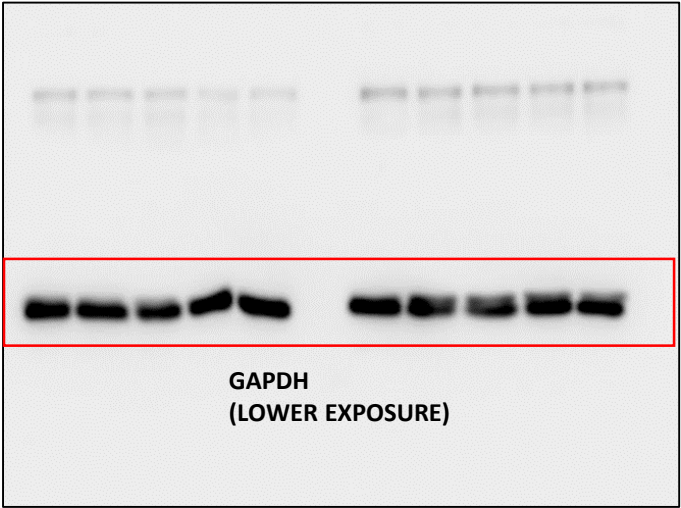

Supplement: Supplementary file 2 — Source Data Fig. 1 [file 44318_2023_18_MOESM2_ESM.zip › Figure 1/Fig 1E/Blot Fig 1E.pdf]

Figure 1

F

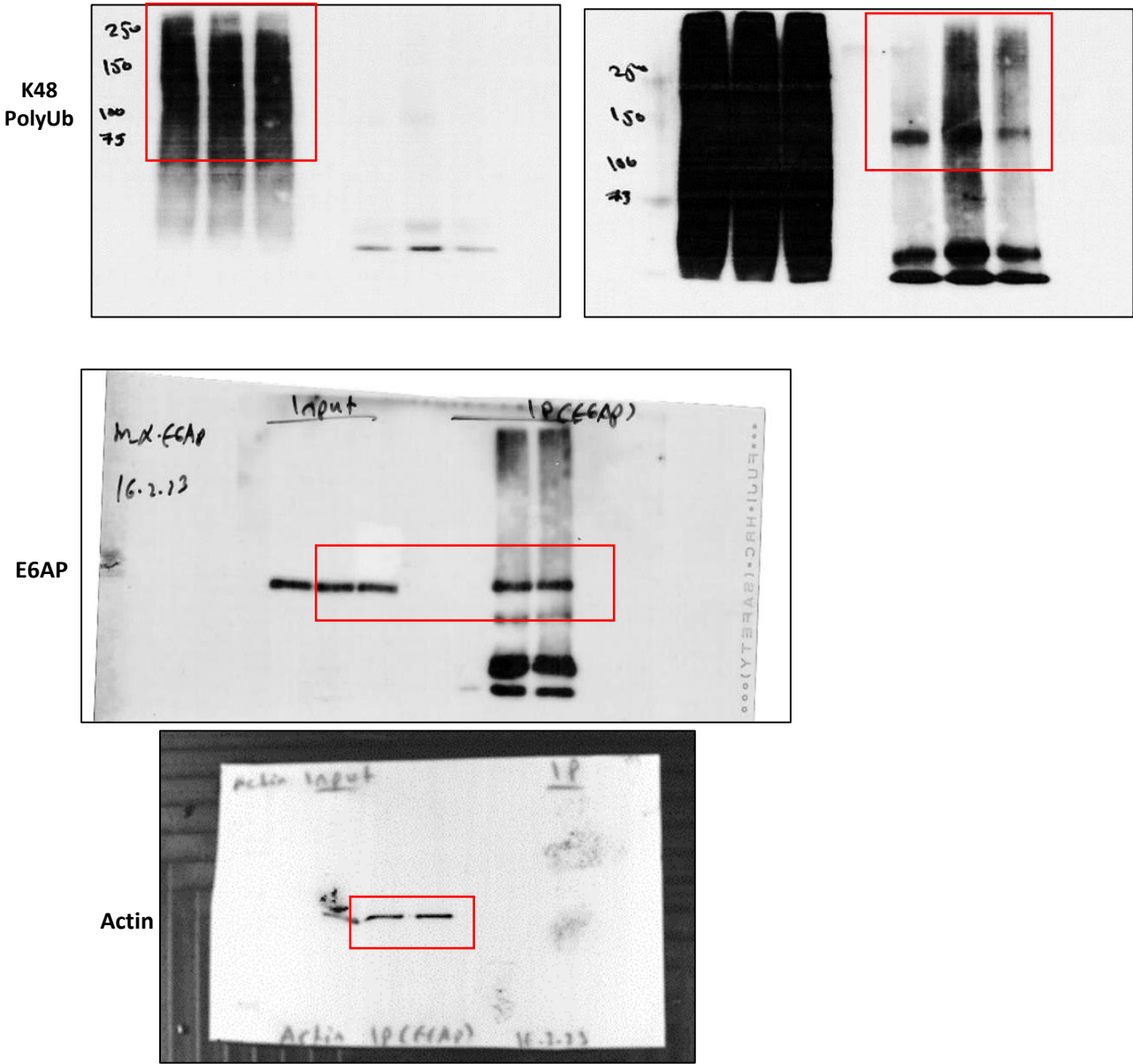

Supplement: Supplementary file 2 — Source Data Fig. 1 [file 44318_2023_18_MOESM2_ESM.zip › Figure 1/Fig 1F/Blot Fig 1F.pdf]

Figure 1

G

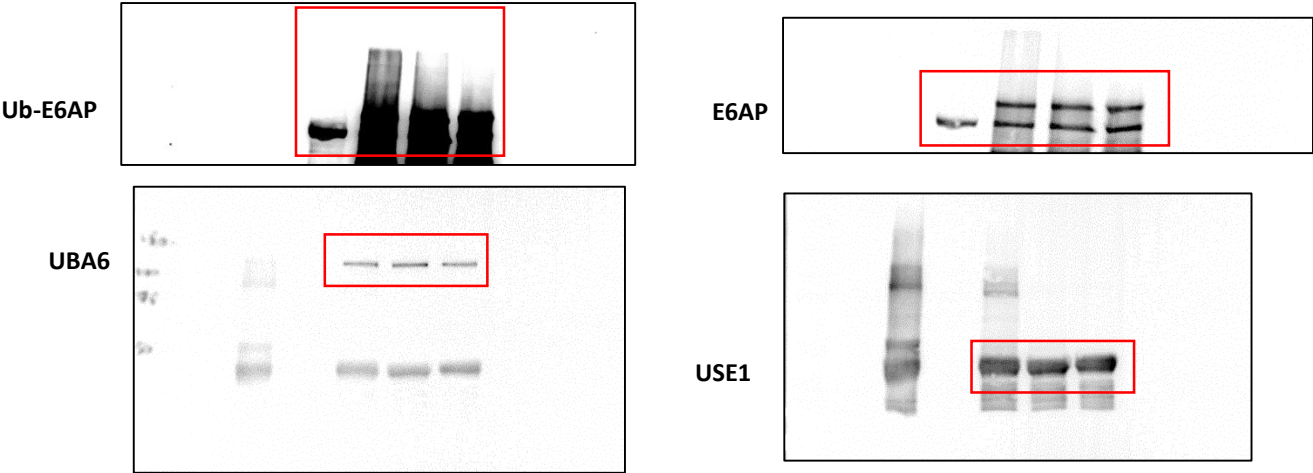

Supplement: Supplementary file 2 — Source Data Fig. 1 [file 44318_2023_18_MOESM2_ESM.zip › Figure 1/Fig 1G/Blot Fig 1G.pdf]

Figure 2

B

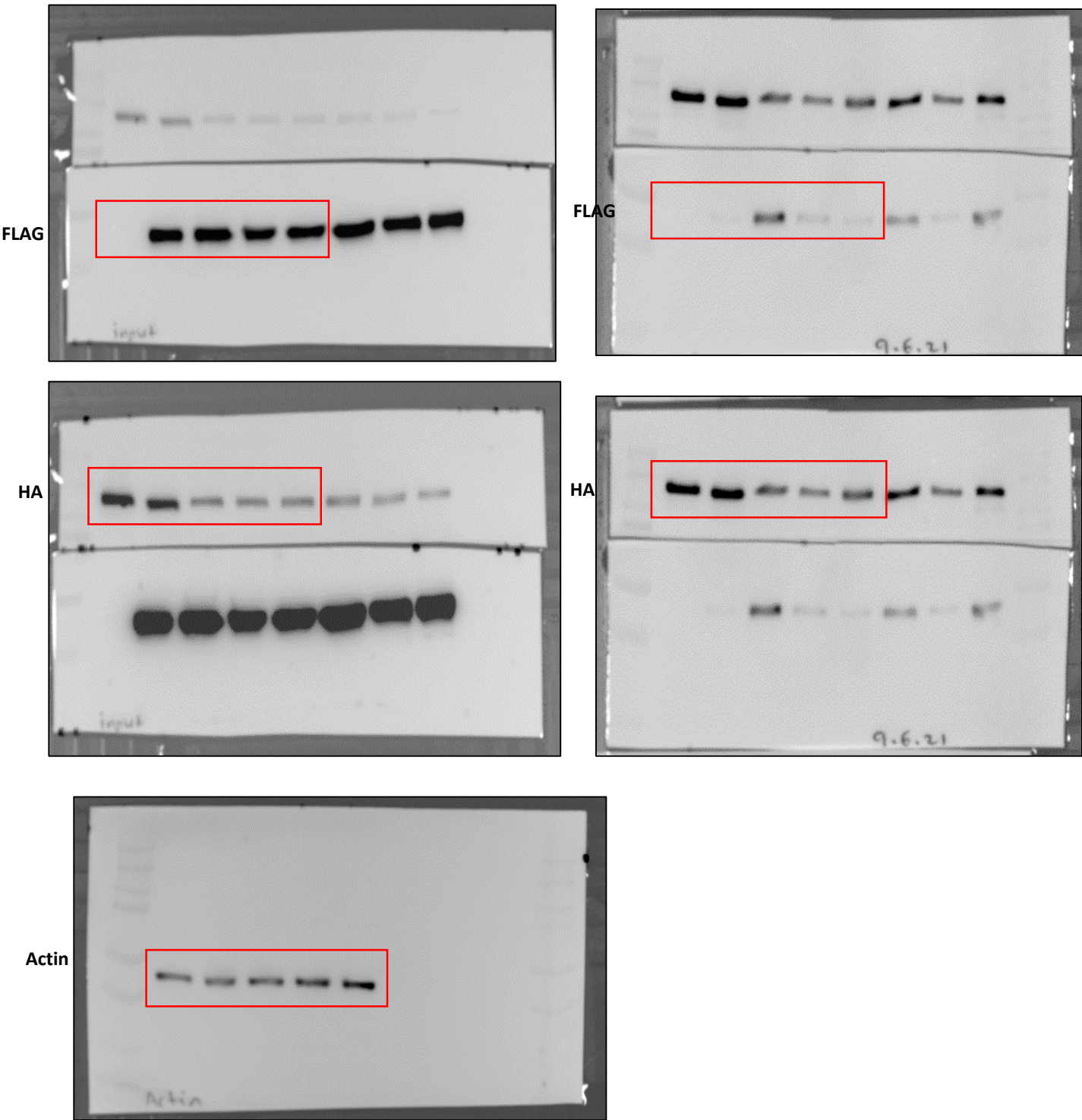

Supplement: Supplementary file 3 — Source Data Fig. 2 [file 44318_2023_18_MOESM3_ESM.zip › Figure 2/Fig 2B/Blot Fig 2B.pdf]

Figure 2

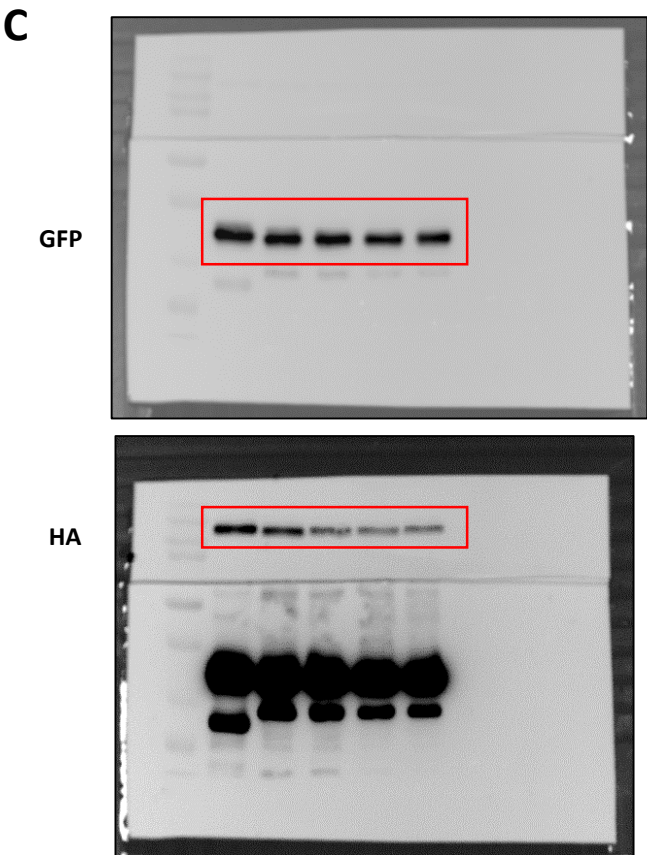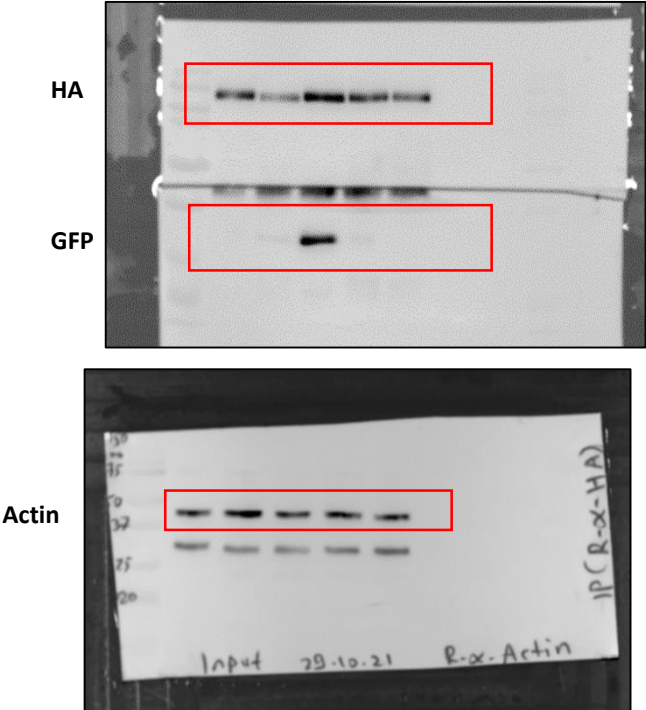

Supplement: Supplementary file 3 — Source Data Fig. 2 [file 44318_2023_18_MOESM3_ESM.zip › Figure 2/Fig 2C/Blot Fig 2C.pdf]

Figure 2

D

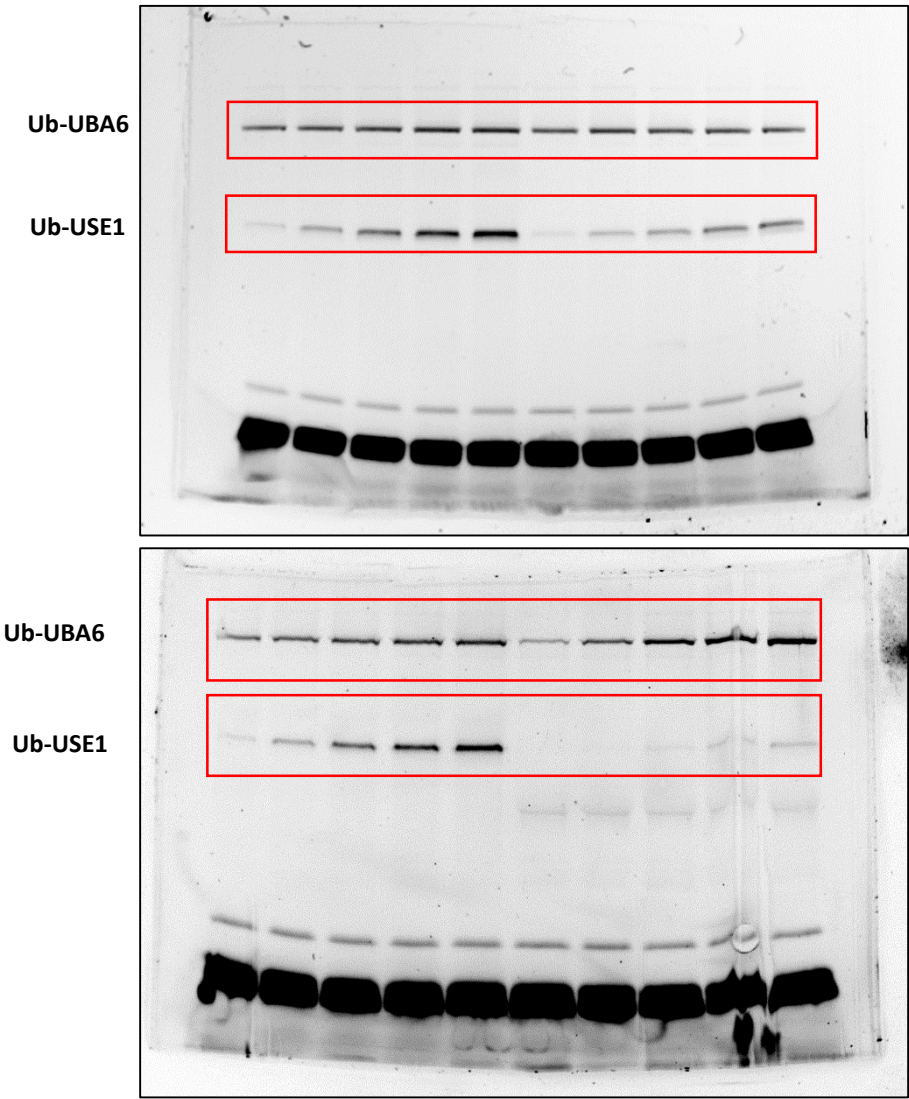

Supplement: Supplementary file 3 — Source Data Fig. 2 [file 44318_2023_18_MOESM3_ESM.zip › Figure 2/Fig 2D/Blot Fig 2D.pdf]

Figure 3

A

GFP

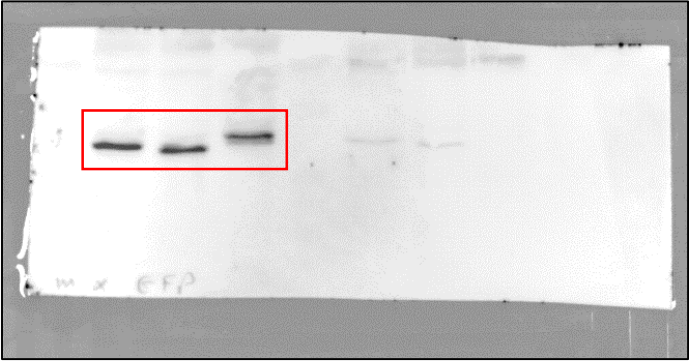

GFP

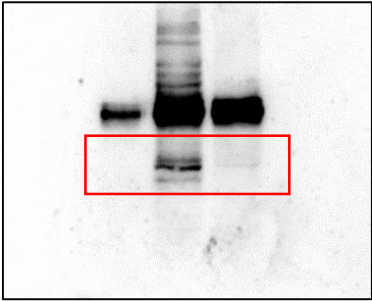

Actin

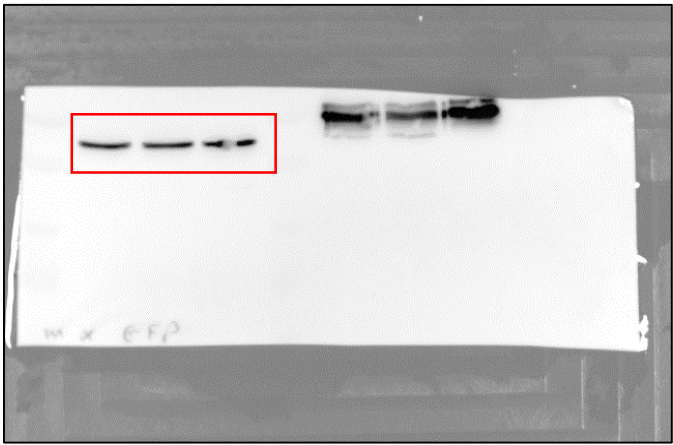

UBA6

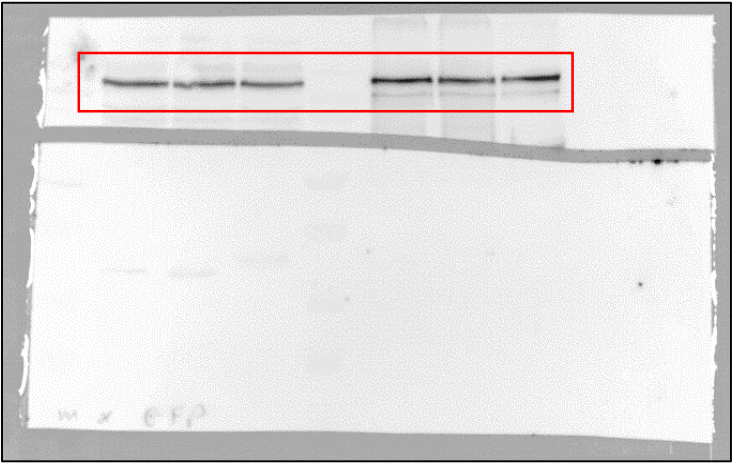

Supplement: Supplementary file 4 — Source Data Fig. 3 [file 44318_2023_18_MOESM4_ESM.zip › Figure 3/Fig 3A/Blot Fig 3A.pdf]

Figure 3

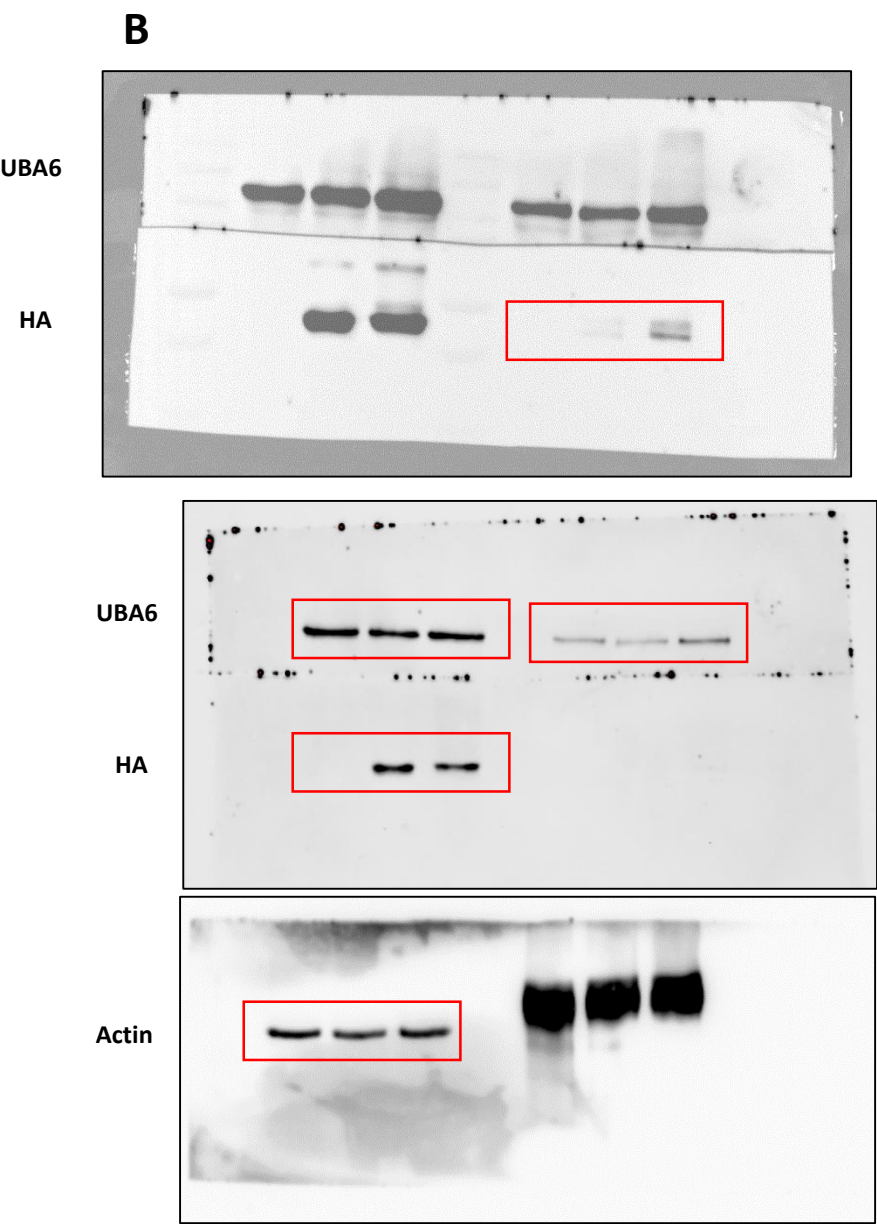

Supplement: Supplementary file 4 — Source Data Fig. 3 [file 44318_2023_18_MOESM4_ESM.zip › Figure 3/Fig 3B/Blot Fig 3B.pdf]

Figure 3  
C

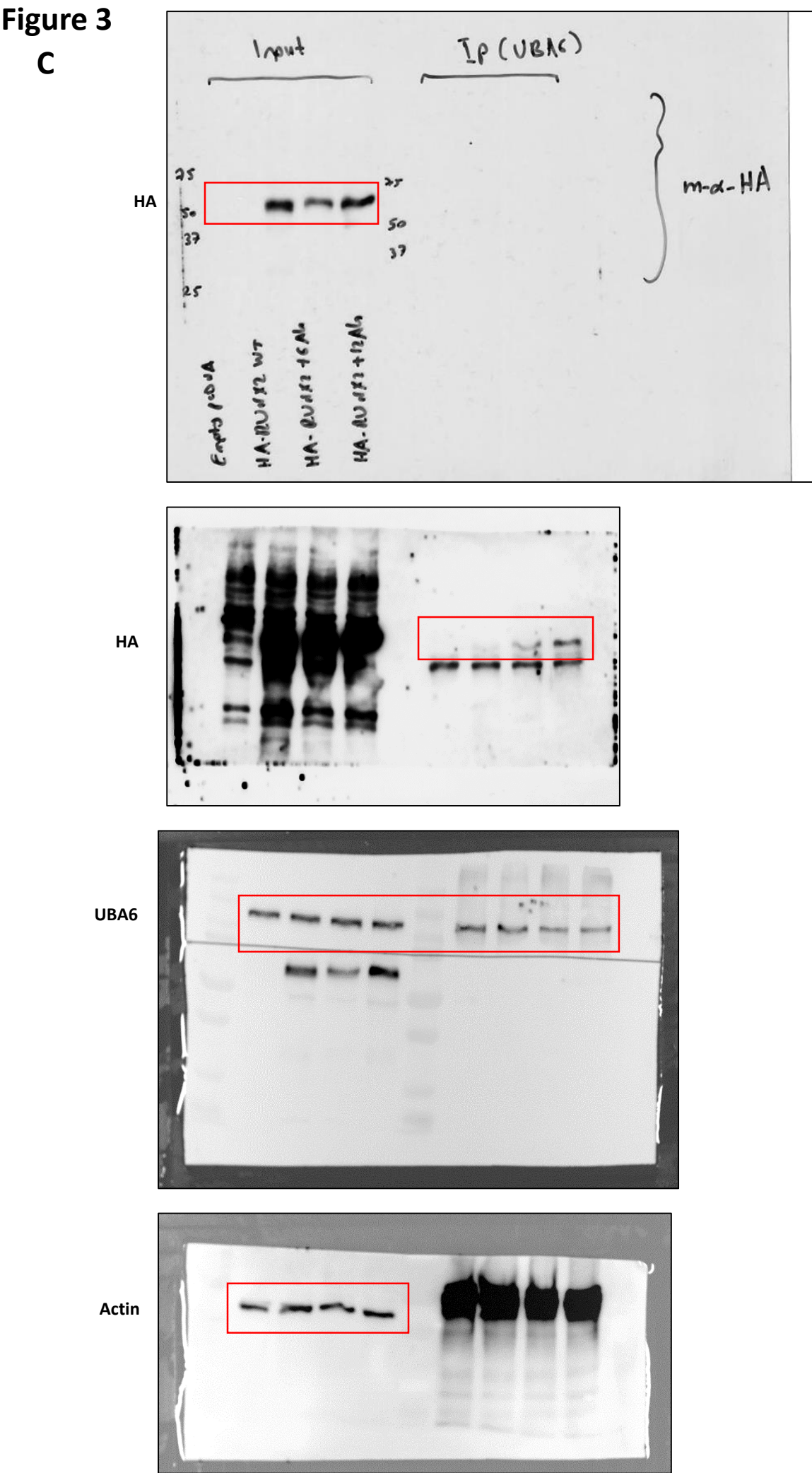

Supplement: Supplementary file 4 — Source Data Fig. 3 [file 44318_2023_18_MOESM4_ESM.zip › Figure 3/Fig 3C/Blot Fig 3C.pdf]

Figure 3

D

UBA6

HA

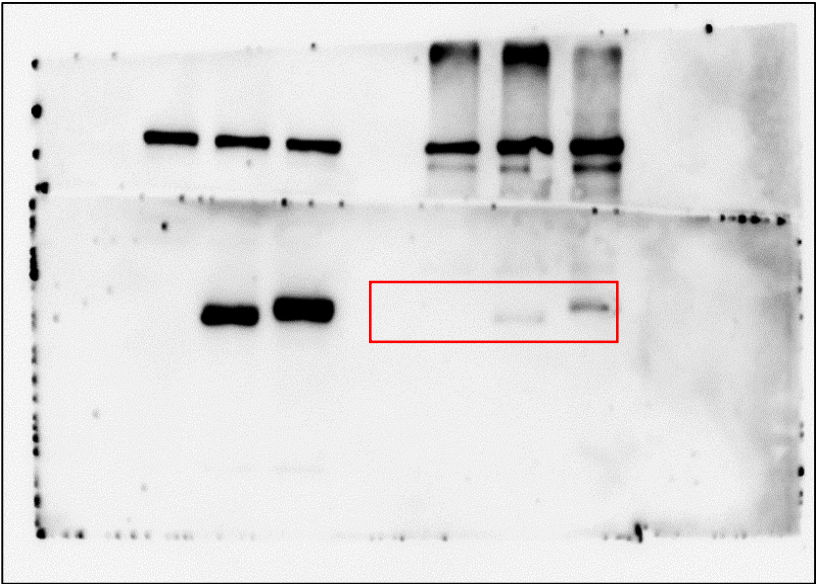

UBA6

HA

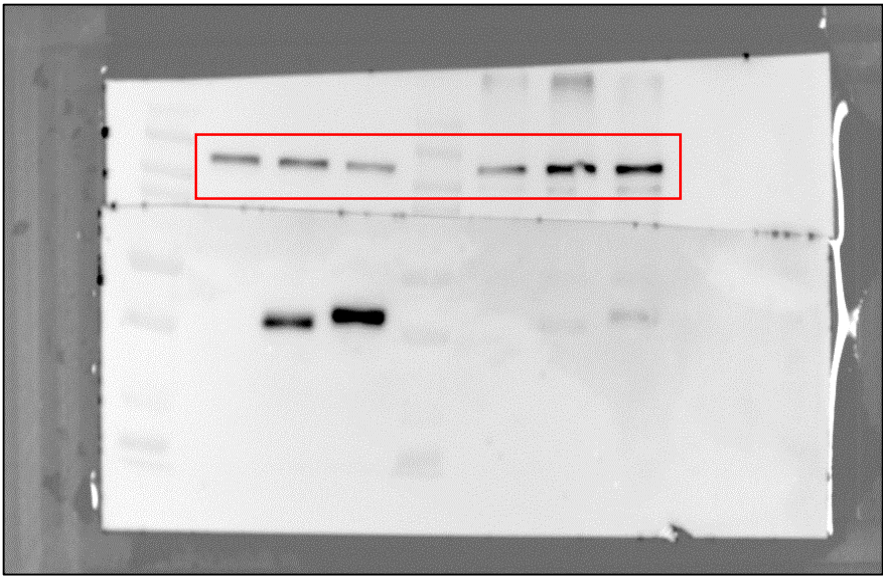

HA

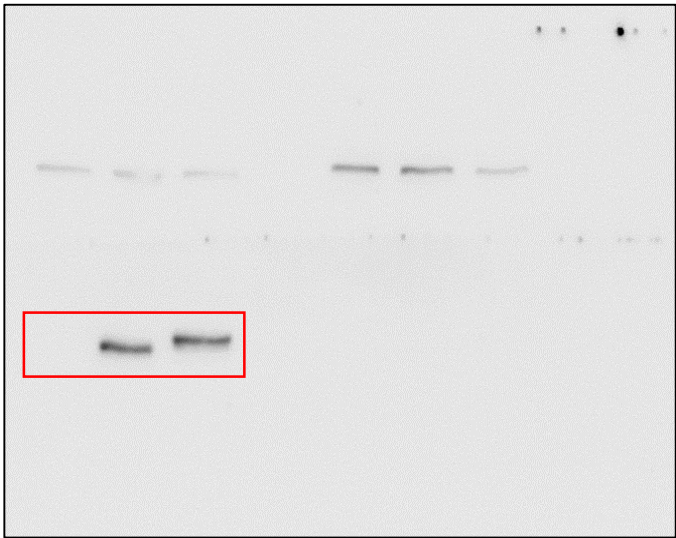

Actin

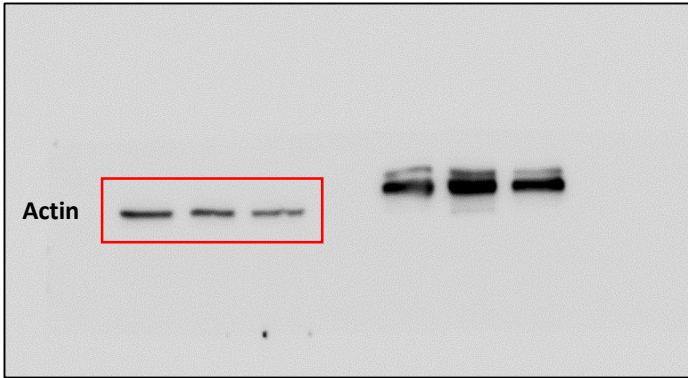

Supplement: Supplementary file 4 — Source Data Fig. 3 [file 44318_2023_18_MOESM4_ESM.zip › Figure 3/Fig 3D/Blot Fig 3D.pdf]

Figure 3

E

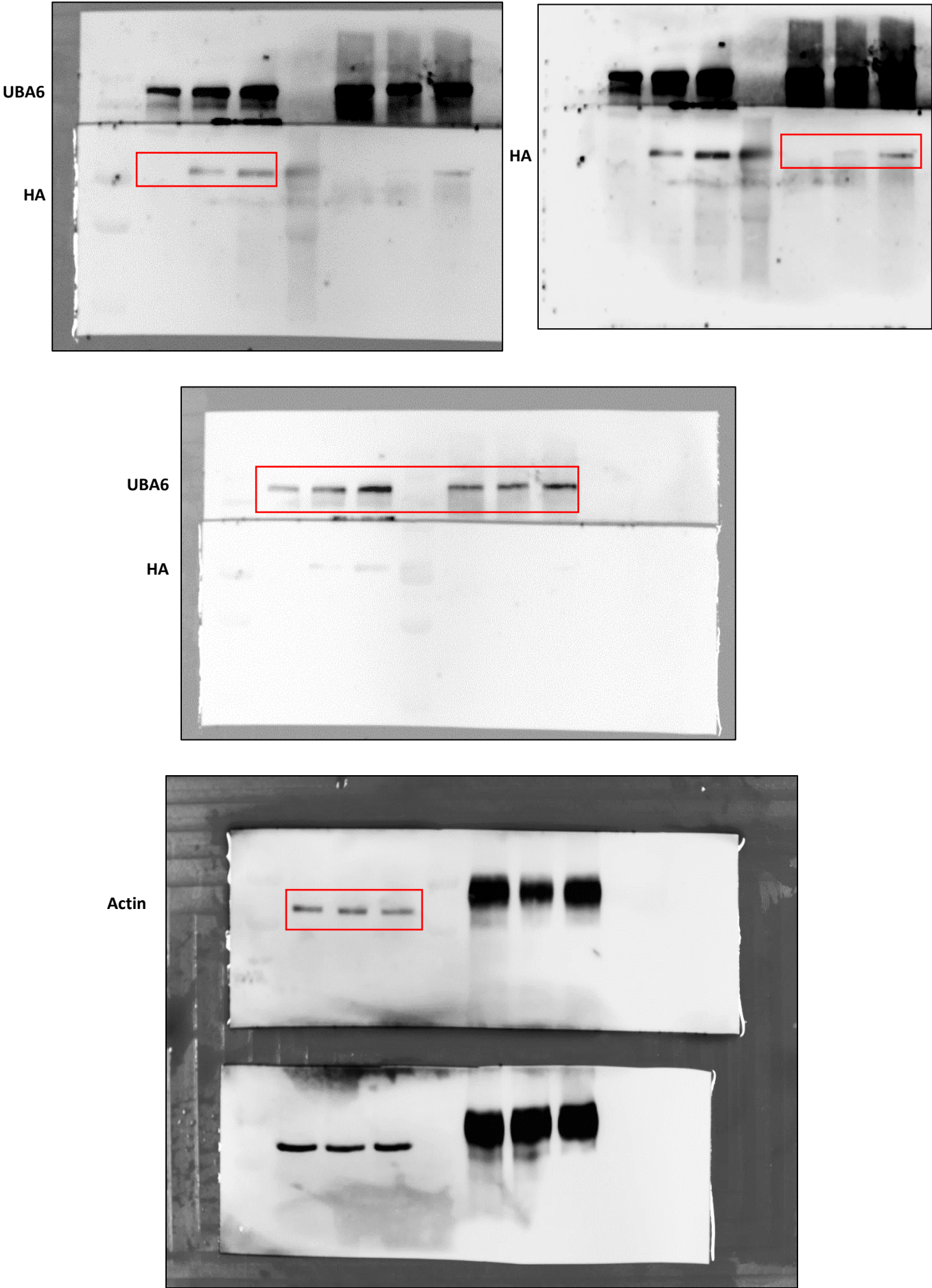

Supplement: Supplementary file 4 — Source Data Fig. 3 [file 44318_2023_18_MOESM4_ESM.zip › Figure 3/Fig 3E/Blot Fig 3E.pdf]

Figure 3

F

HA

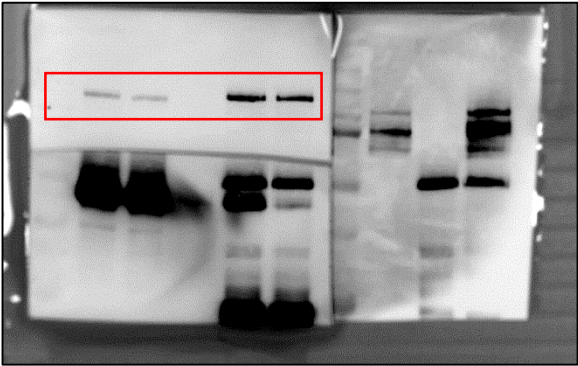

FLAG

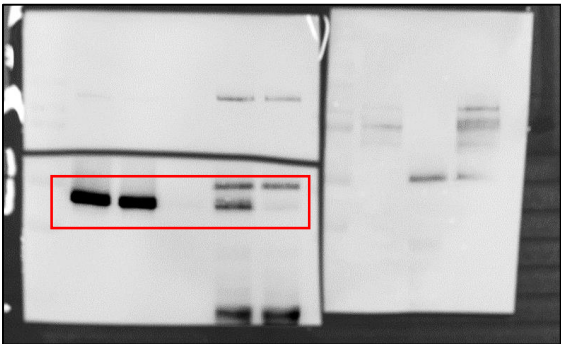

PHOX2B

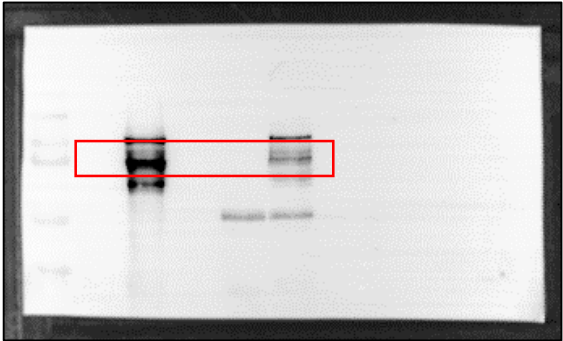

Supplement: Supplementary file 4 — Source Data Fig. 3 [file 44318_2023_18_MOESM4_ESM.zip › Figure 3/Fig 3F/Blot Fig 3F.pdf]

Figure 3

G

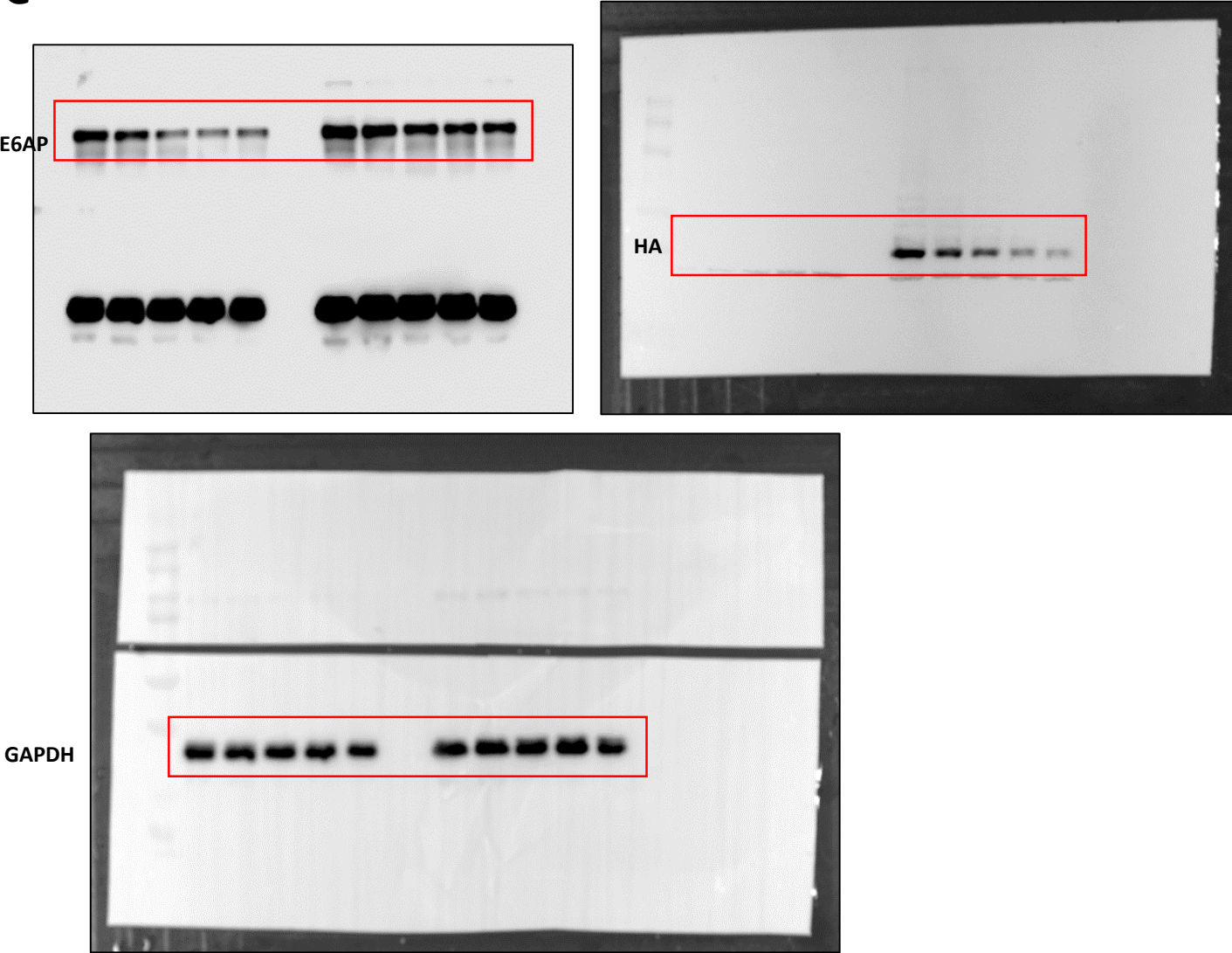

Supplement: Supplementary file 4 — Source Data Fig. 3 [file 44318_2023_18_MOESM4_ESM.zip › Figure 3/Fig 3G/Blot Fig 3G.pdf]

Figure 3

I

K48  
PolyUb

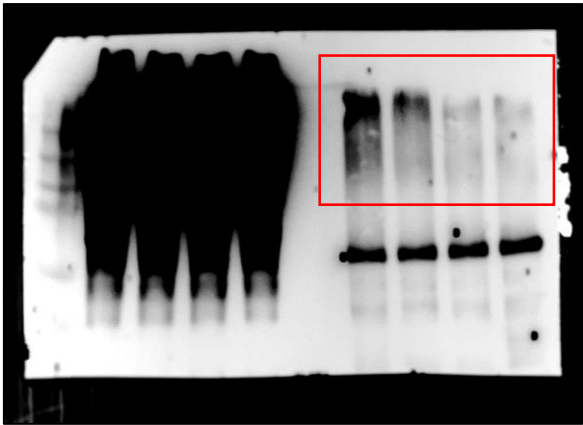

K48  
PolyUb

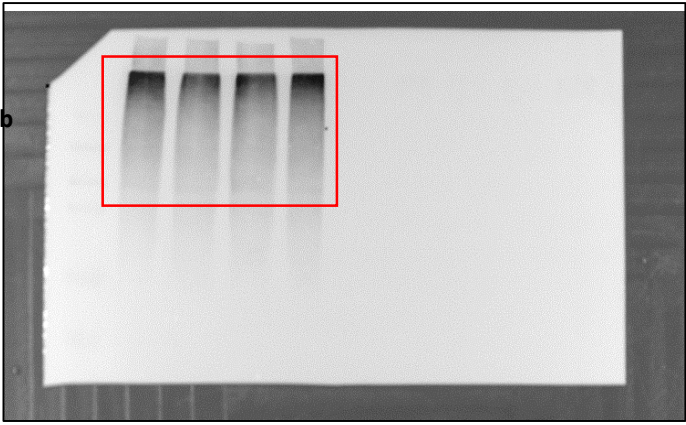

E6AP

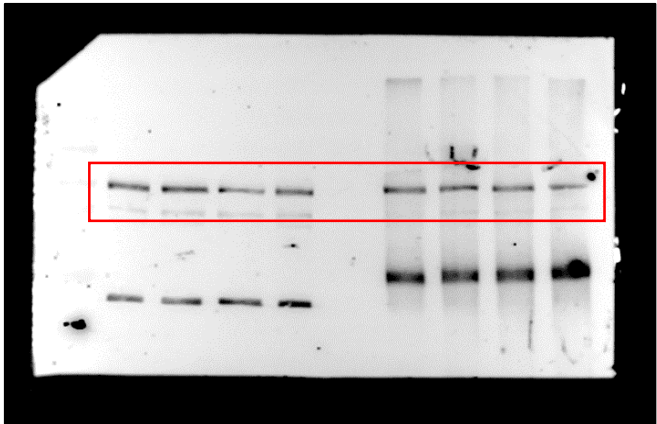

UBA6

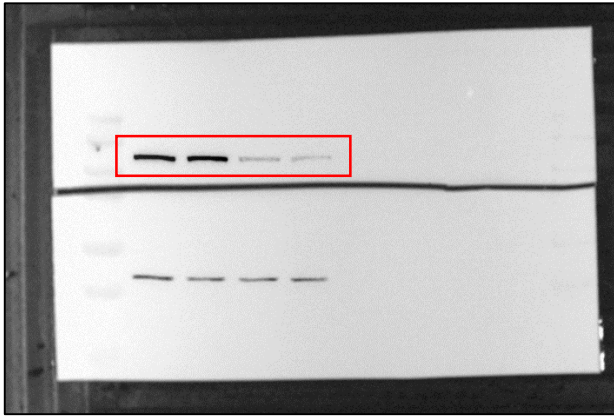

PHOX2B

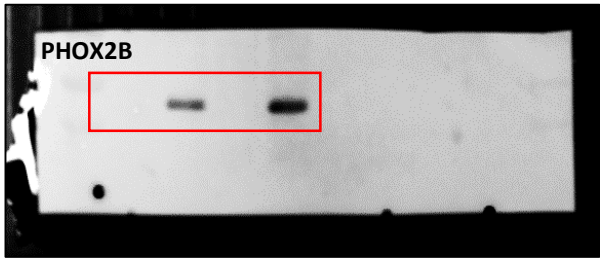

Actin

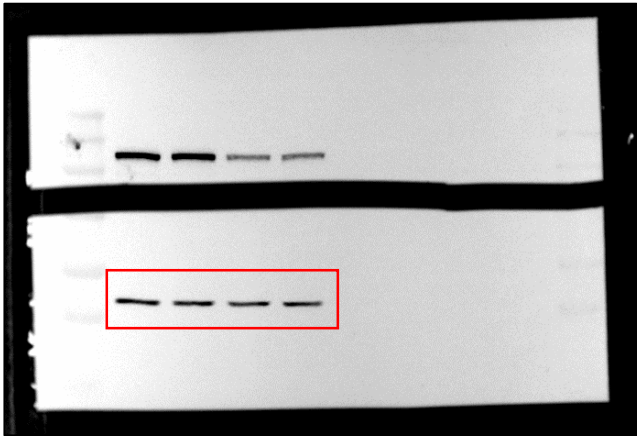

Supplement: Supplementary file 4 — Source Data Fig. 3 [file 44318_2023_18_MOESM4_ESM.zip › Figure 3/Fig 3I/Blot Fig 3I.pdf]

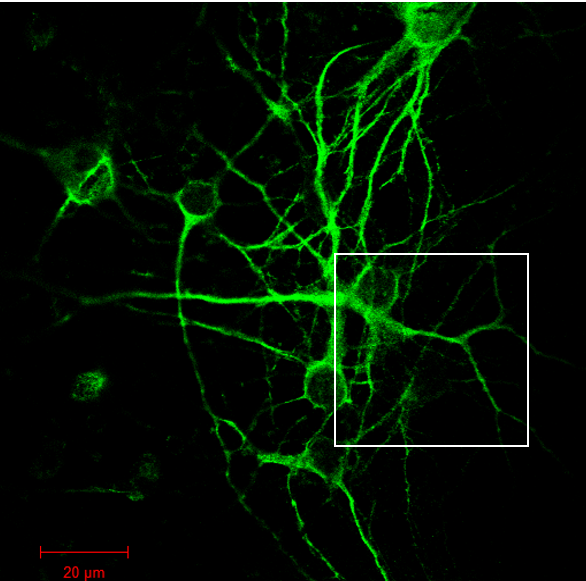

Supplement: Supplementary file 5 — Source Data Fig. 4 [file 44318_2023_18_MOESM5_ESM.zip › Figure 4/Fig 4A/Image 4A MAP2.tif]

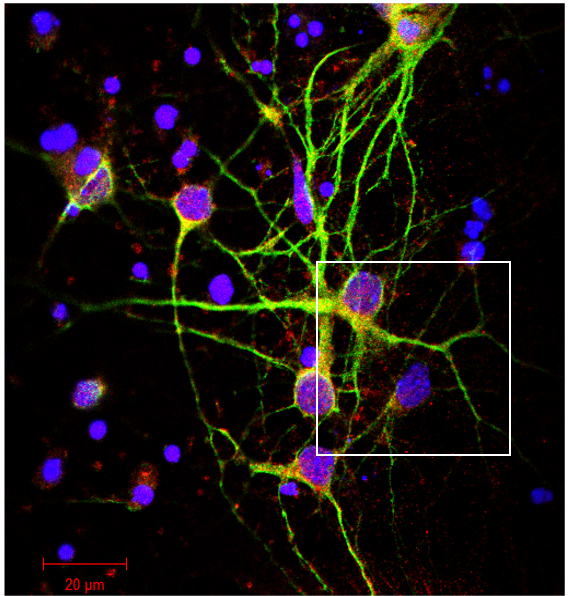

Supplement: Supplementary file 5 — Source Data Fig. 4 [file 44318_2023_18_MOESM5_ESM.zip › Figure 4/Fig 4A/Image 4A Merged.tif]

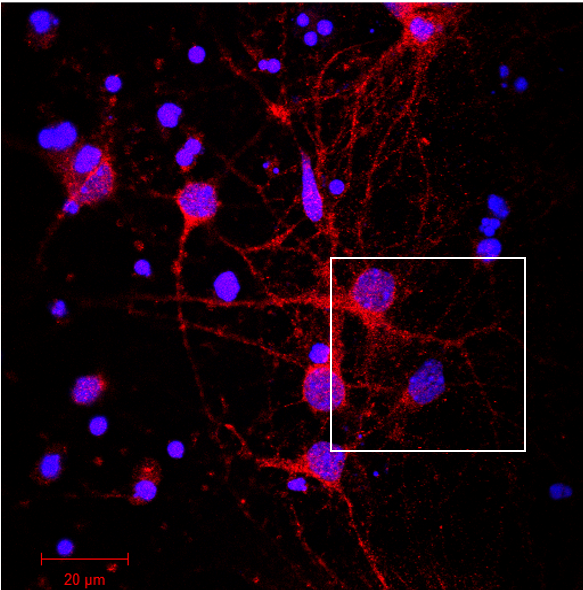

Supplement: Supplementary file 5 — Source Data Fig. 4 [file 44318_2023_18_MOESM5_ESM.zip › Figure 4/Fig 4A/Image 4A UBA6 Nuclei.tif]

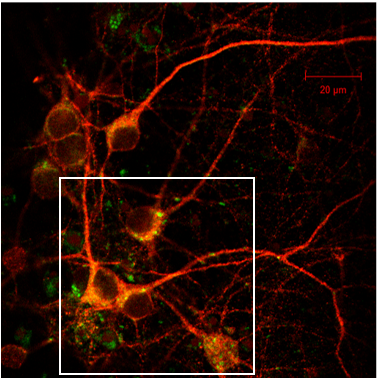

Supplement: Supplementary file 5 — Source Data Fig. 4 [file 44318_2023_18_MOESM5_ESM.zip › Figure 4/Fig 4B/Image 4B GFP + 13 Ala MAP2.tif]

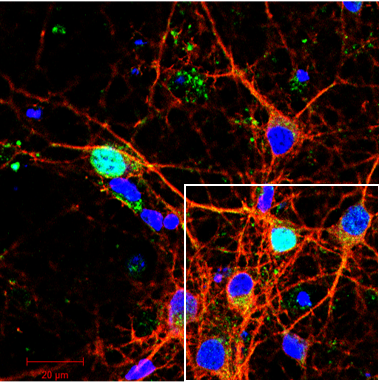

Supplement: Supplementary file 5 — Source Data Fig. 4 [file 44318_2023_18_MOESM5_ESM.zip › Figure 4/Fig 4B/Image 4B GFP +13 Ala Nuclei TAU.tif]

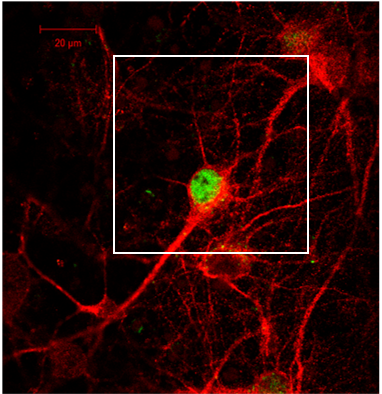

Supplement: Supplementary file 5 — Source Data Fig. 4 [file 44318_2023_18_MOESM5_ESM.zip › Figure 4/Fig 4B/Image 4B GFP WT MAP2.tif]

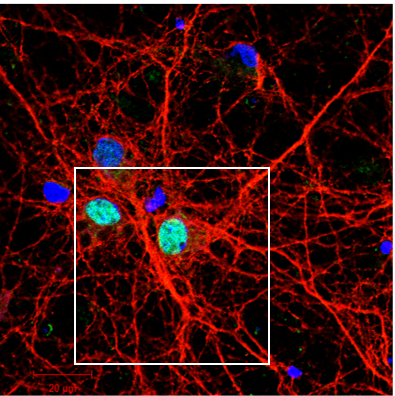

Supplement: Supplementary file 5 — Source Data Fig. 4 [file 44318_2023_18_MOESM5_ESM.zip › Figure 4/Fig 4B/Image 4B GFP WT Nuclei TAU.tif]

Figure 4

C

PHOX2B

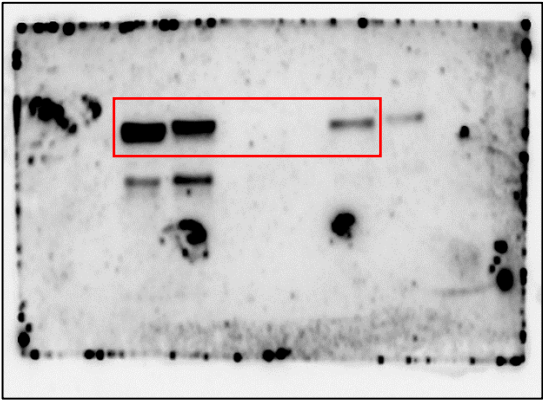

GAPDH

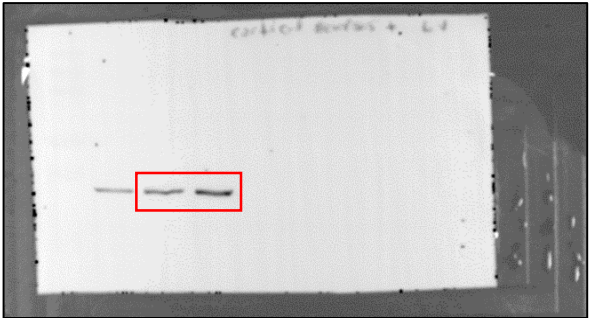

Supplement: Supplementary file 5 — Source Data Fig. 4 [file 44318_2023_18_MOESM5_ESM.zip › Figure 4/Fig 4C/Blot Fig 4C.pdf]

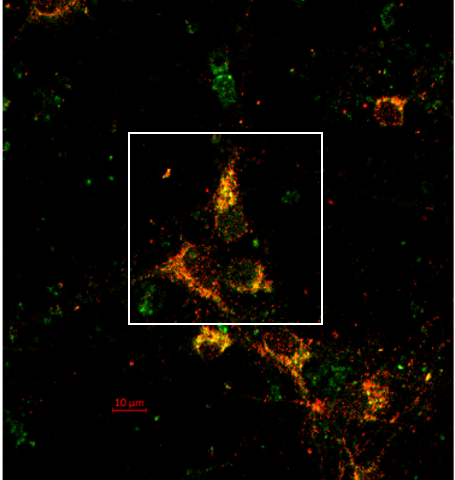

Supplement: Supplementary file 5 — Source Data Fig. 4 [file 44318_2023_18_MOESM5_ESM.zip › Figure 4/Fig 4D/Image 4D PHOX2B + 13 Ala UBA6.tif]

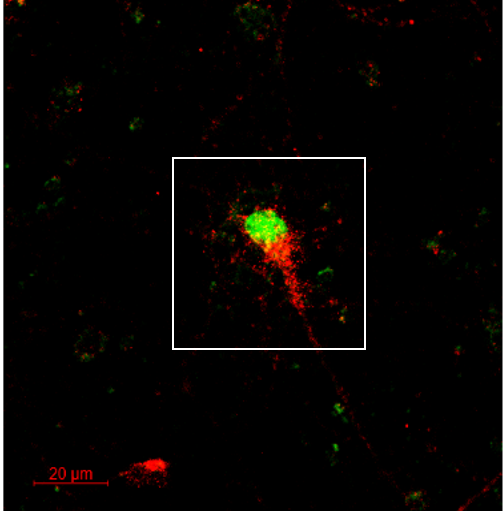

Supplement: Supplementary file 5 — Source Data Fig. 4 [file 44318_2023_18_MOESM5_ESM.zip › Figure 4/Fig 4D/Image 4D PHOX2B + 7 Ala UBA6.tif]

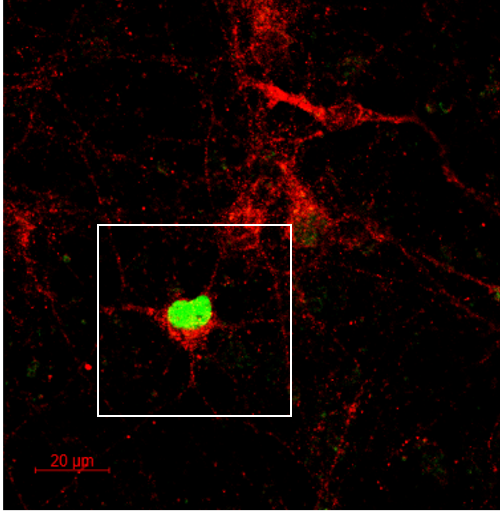

Supplement: Supplementary file 5 — Source Data Fig. 4 [file 44318_2023_18_MOESM5_ESM.zip › Figure 4/Fig 4D/Image 4D WT PHOX2B UBA6.tif]

Figure 4

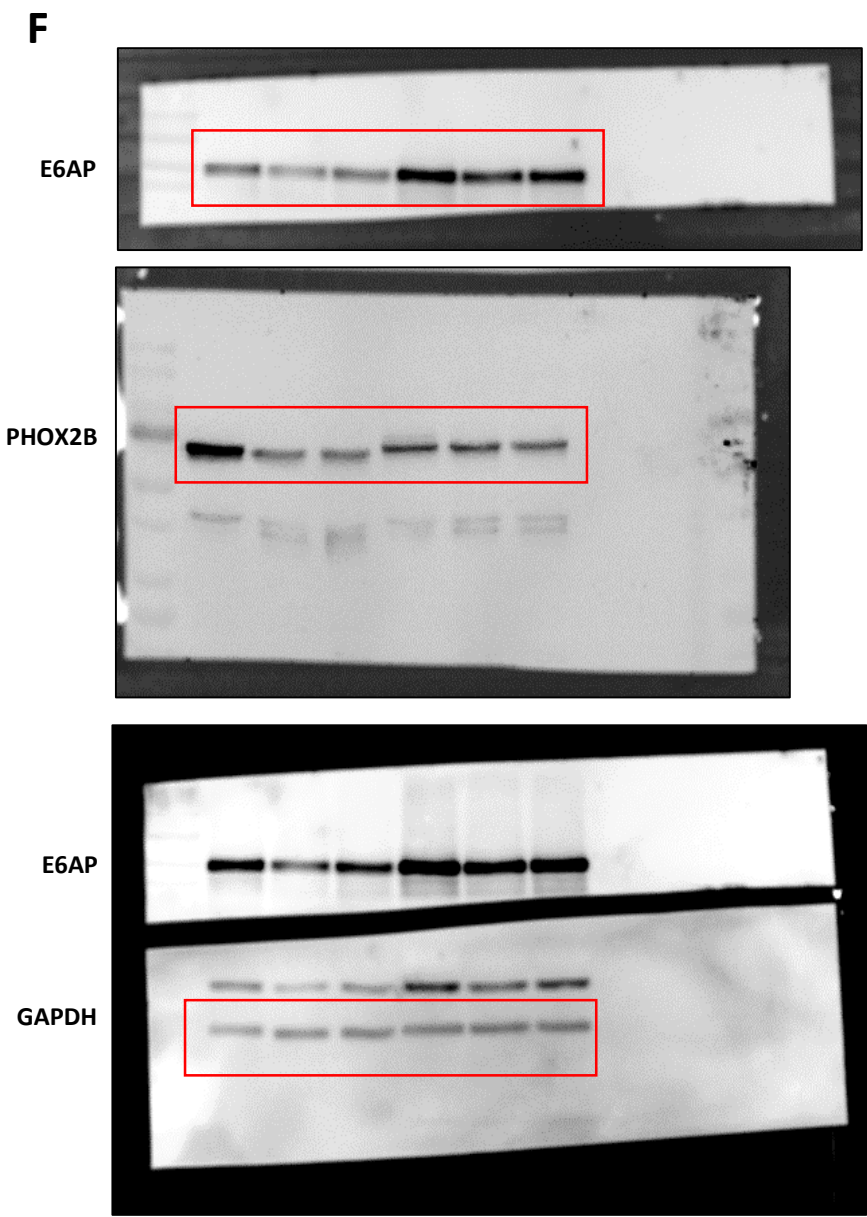

Supplement: Supplementary file 5 — Source Data Fig. 4 [file 44318_2023_18_MOESM5_ESM.zip › Figure 4/Fig 4F/Blot Fig 4F.pdf]

Figure 4

G

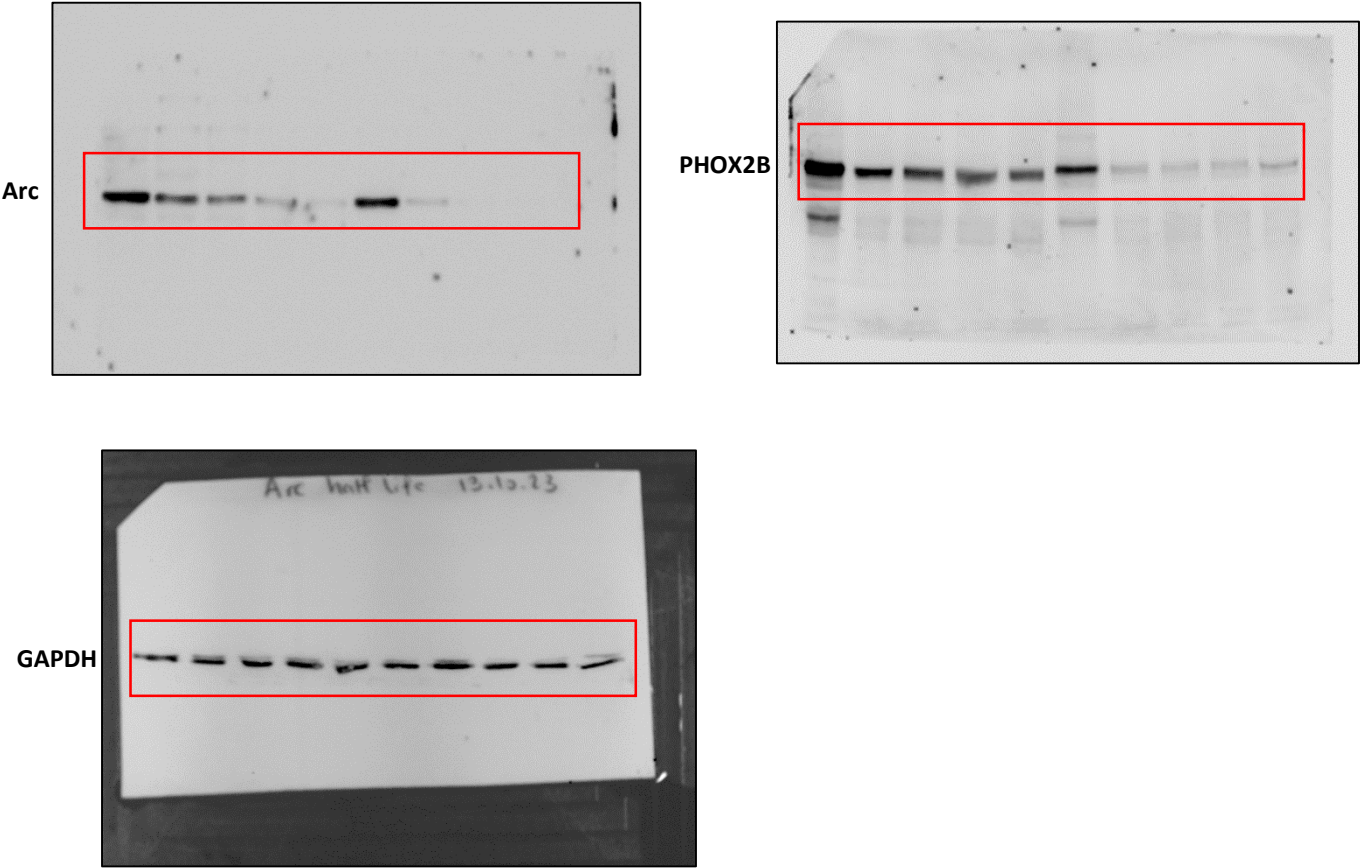

Supplement: Supplementary file 5 — Source Data Fig. 4 [file 44318_2023_18_MOESM5_ESM.zip › Figure 4/Fig 4G/Blot Fig 4G.pdf]

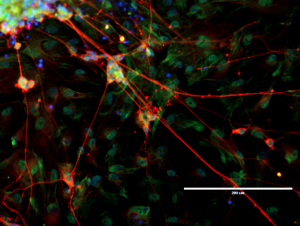

Supplement: Supplementary file 6 — Source Data Fig. 5 [file 44318_2023_18_MOESM6_ESM.zip › Figure 5/Fig 5A/Image 4A 102iCCHS ATOH1 TUB3 Nuclei.tif]

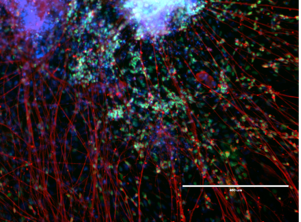

Supplement: Supplementary file 6 — Source Data Fig. 5 [file 44318_2023_18_MOESM6_ESM.zip › Figure 5/Fig 5A/Image 4A 102iCCHS PHOX2B TUB3 Nuclei.tif]

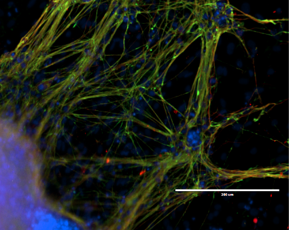

Supplement: Supplementary file 6 — Source Data Fig. 5 [file 44318_2023_18_MOESM6_ESM.zip › Figure 5/Fig 5A/Image 4A 102iCCHS PRPH TUB3 Nuclei.tif]

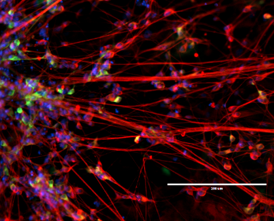

Supplement: Supplementary file 6 — Source Data Fig. 5 [file 44318_2023_18_MOESM6_ESM.zip › Figure 5/Fig 5A/Image 4A 102iCCHS TH TUB3 Nuclei.tif]

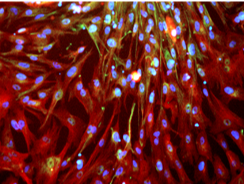

Supplement: Supplementary file 6 — Source Data Fig. 5 [file 44318_2023_18_MOESM6_ESM.zip › Figure 5/Fig 5A/Image 4A 103iCTR ATOH1 TUB3 Nuclei.tif]

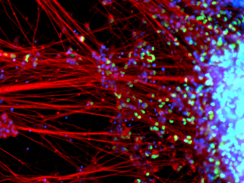

Supplement: Supplementary file 6 — Source Data Fig. 5 [file 44318_2023_18_MOESM6_ESM.zip › Figure 5/Fig 5A/Image 4A 103iCTR PHOX2B TUB3 Nuclei.tif]

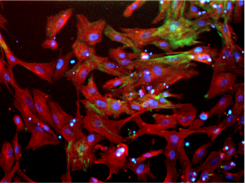

Supplement: Supplementary file 6 — Source Data Fig. 5 [file 44318_2023_18_MOESM6_ESM.zip › Figure 5/Fig 5A/Image 4A 103iCTR PRPH TUB3 Nuclei.tif]

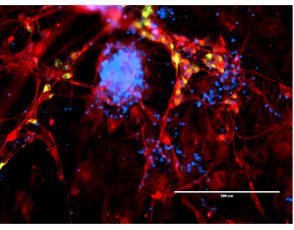

Supplement: Supplementary file 6 — Source Data Fig. 5 [file 44318_2023_18_MOESM6_ESM.zip › Figure 5/Fig 5A/Image 4A 103iCTR TH TUB3 Nuclei.tif]

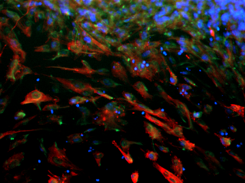

Supplement: Supplementary file 6 — Source Data Fig. 5 [file 44318_2023_18_MOESM6_ESM.zip › Figure 5/Fig 5A/Image 4A 104iCCHS ATOH1 TUB3 Nuclei.tif]

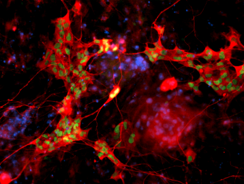

Supplement: Supplementary file 6 — Source Data Fig. 5 [file 44318_2023_18_MOESM6_ESM.zip › Figure 5/Fig 5A/Image 4A 104iCCHS PHOX2B TUB3 Nuclei.tif]

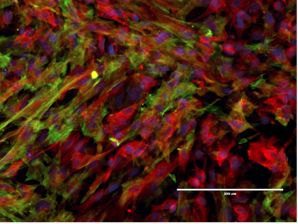

Supplement: Supplementary file 6 — Source Data Fig. 5 [file 44318_2023_18_MOESM6_ESM.zip › Figure 5/Fig 5A/Image 4A 104iCCHS PRPH TUB3 Nuclei.tif]

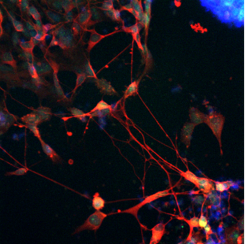

Supplement: Supplementary file 6 — Source Data Fig. 5 [file 44318_2023_18_MOESM6_ESM.zip › Figure 5/Fig 5A/Image 4A 104iCCHS TH TUB3 Nuclei.tif]

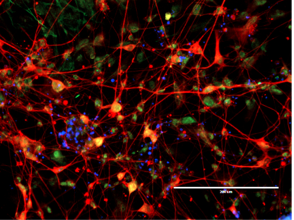

Supplement: Supplementary file 6 — Source Data Fig. 5 [file 44318_2023_18_MOESM6_ESM.zip › Figure 5/Fig 5A/Image 4A 105iCTR ATOH1 TUB3 Nuclei.tif]

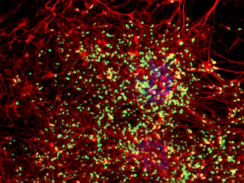

Supplement: Supplementary file 6 — Source Data Fig. 5 [file 44318_2023_18_MOESM6_ESM.zip › Figure 5/Fig 5A/Image 4A 105iCTR PHOX2B TUB3 Nuclei.tif]

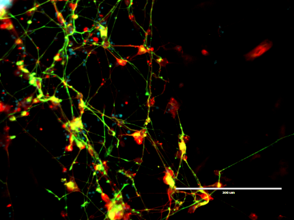

Supplement: Supplementary file 6 — Source Data Fig. 5 [file 44318_2023_18_MOESM6_ESM.zip › Figure 5/Fig 5A/Image 4A 105iCTR PRPH TUB3 Nuclei.tif]

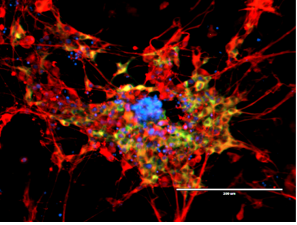

Supplement: Supplementary file 6 — Source Data Fig. 5 [file 44318_2023_18_MOESM6_ESM.zip › Figure 5/Fig 5A/Image 4A 105iCTR TH TUB3 Nuclei.tif]

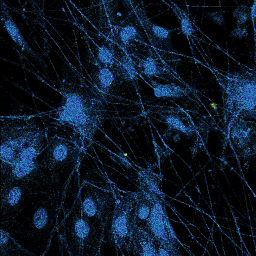

Supplement: Supplementary file 6 — Source Data Fig. 5 [file 44318_2023_18_MOESM6_ESM.zip › Figure 5/Fig 5C/Image 5C 102iCCHS 20.25.tif]

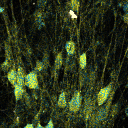

Supplement: Supplementary file 6 — Source Data Fig. 5 [file 44318_2023_18_MOESM6_ESM.zip › Figure 5/Fig 5C/Image 5C 105iCTR 20.20.tif]

Figure 5

D

E6AP

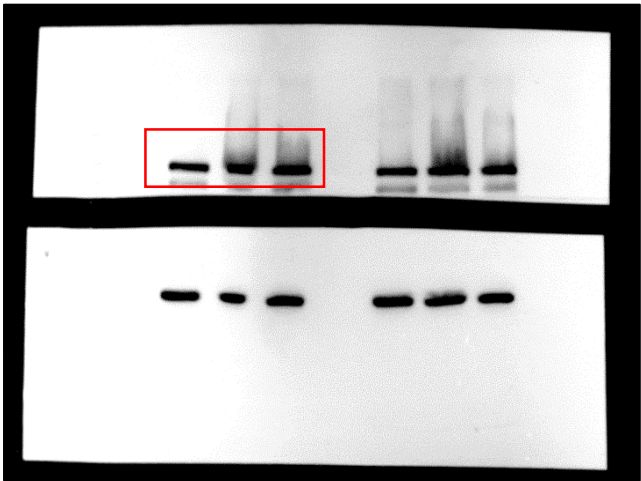

Actin

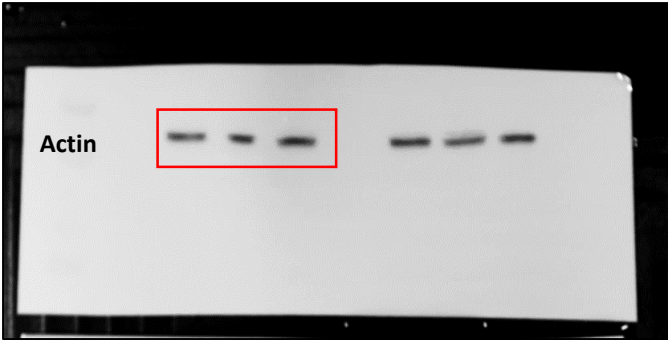

Supplement: Supplementary file 6 — Source Data Fig. 5 [file 44318_2023_18_MOESM6_ESM.zip › Figure 5/Fig 5D/Blot Fig 5D.pdf]

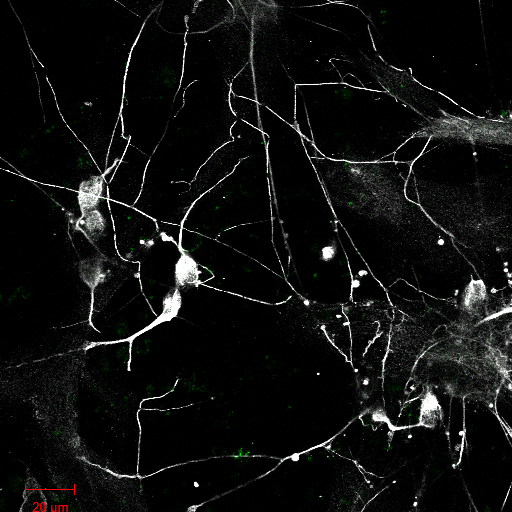

Supplement: Supplementary file 6 — Source Data Fig. 5 [file 44318_2023_18_MOESM6_ESM.zip › Figure 5/Fig 5F/Image Fig 5F CCHS neurons UBA6 cDNA.tif]

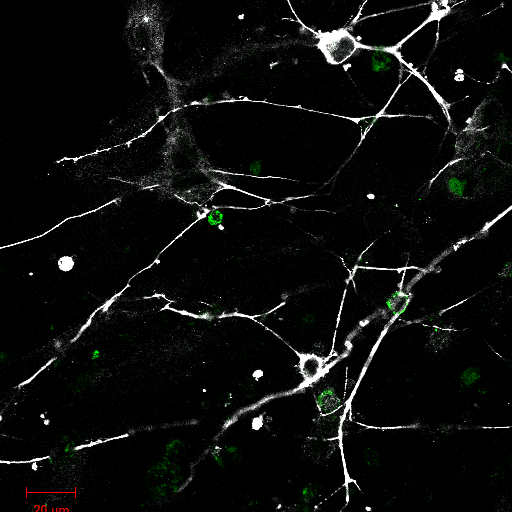

Supplement: Supplementary file 6 — Source Data Fig. 5 [file 44318_2023_18_MOESM6_ESM.zip › Figure 5/Fig 5F/Image Fig 5F CCHS neurons.tif]

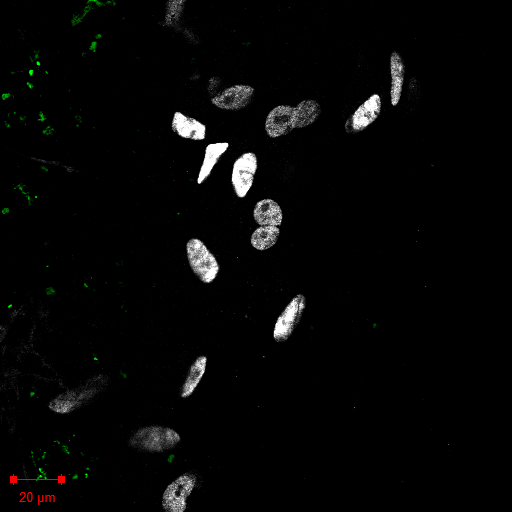

Supplement: Supplementary file 6 — Source Data Fig. 5 [file 44318_2023_18_MOESM6_ESM.zip › Figure 5/Fig 5G/Image Fig 5G CCHS neurons UBA6 cDNA.tif]

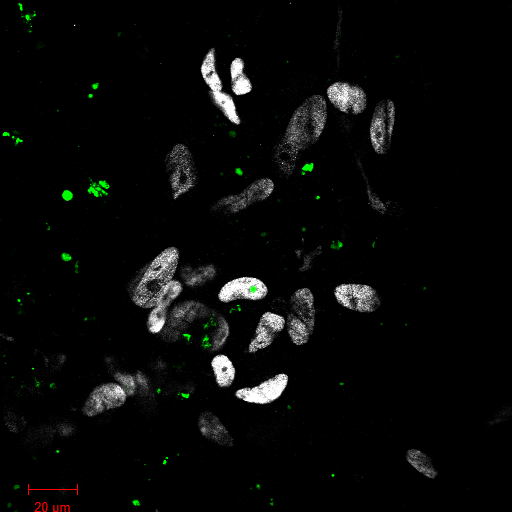

Supplement: Supplementary file 6 — Source Data Fig. 5 [file 44318_2023_18_MOESM6_ESM.zip › Figure 5/Fig 5G/Image Fig 5G CCHS neurons.tif]

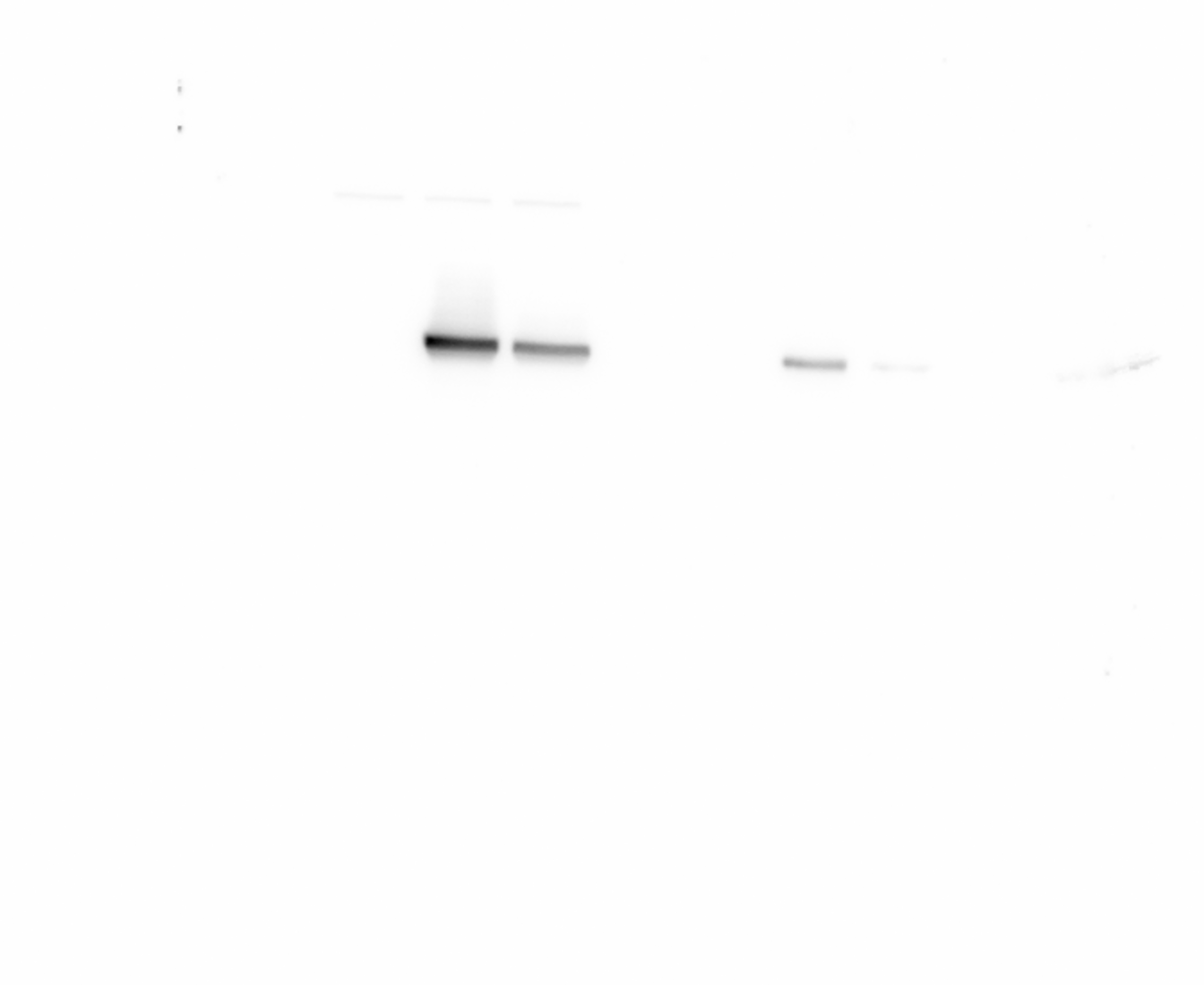

Supplement: Supplementary file 7 — EV and Appendix Figure Source Data [file 44318_2023_18_MOESM7_ESM.zip › FIGEV1D_FLAG_SE.Tif]
