# Supplementary material for: Disease-associated polyalanine expansion mutations impair UBA6-dependent ubiquitination
Source: EMBO J. 2024 Jan 2;43(2):5. doi: 10.1038/s44318-023-00018-9 (PMC10897158; doi:10.1038/s44318-023-00018-9)
Supplement: Supplementary file 9 — Expanded View Figures [file 44318_2023_18_MOESM9_ESM.pdf]

Expanded View Figures

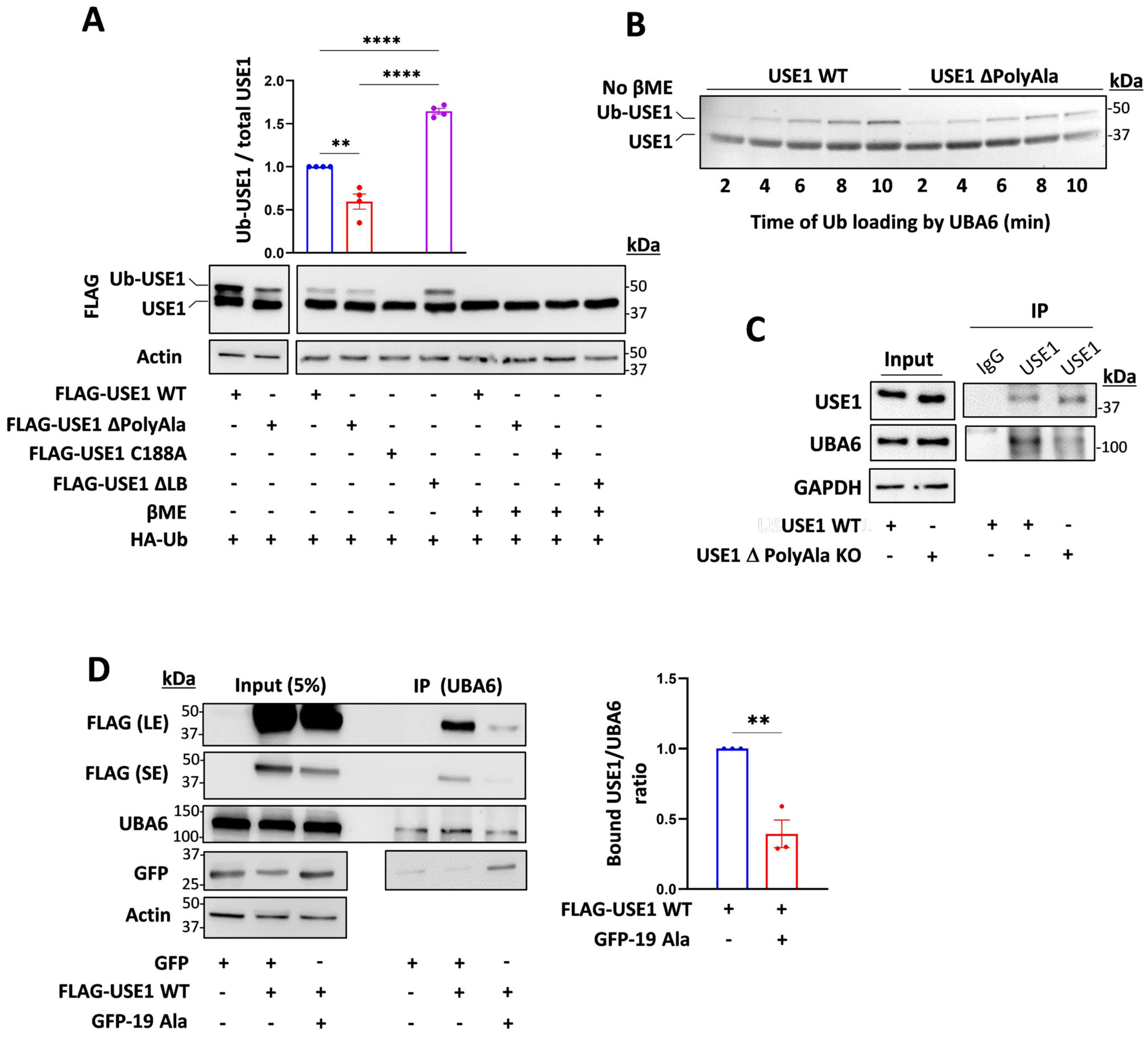

Figure EV1. Polyalanine stretches regulate UBA6-USE1 interaction and ubiquitin transfer.

(**A**) FLAG-WT USE1, FLAG-USE1 ΔPolyAla, FLAG-USE1 C188A, and FLAG-USE1 ΔLB were co-expressed with HA-Ub in HEK293T cells. Cell lysates were incubated with or without β mercaptoethanol (βME) and analyzed for ubiquitin loading. Results are normalized to control WT USE1. $n = 4$ biological replicates. (**B**) A representative blot for time-dependent in vitro ubiquitin loading of WT and ΔPolyAla USE1 by UBA6 (quantification is presented in Fig. 1C). (**C**) WT and ΔPolyAla KO cells were treated with the cross-linker formaldehyde and cell lysates were immunoprecipitated with anti-USE1 or control IgG antibodies. Immunocomplexes were analyzed with anti-USE1 and anti-UBA6 antibodies. (**D**) FLAG-WT USE1, empty GFP and GFP-polyAla (19Ala) constructs were transfected into HEK293T cells. Cell lysates were immunoprecipitated with anti-UBA6 antibodies and the immunocomplexes were analyzed with anti-FLAG, anti-UBA6 and anti-GFP antibodies. The bound USE1/UBA6 ratio is shown. $n = 3$ biological replicates. Data information: Data points in (**A**, **D**) represent mean ± s.e.m. *P* values were calculated by one-way ANOVA Tukey's test (**A**) or paired 2-tailed *t* test (**D**). $^{**}P < 0.01$, $^{****}P < 0.0001$. Source data are available online for this figure.

© The Author(s)

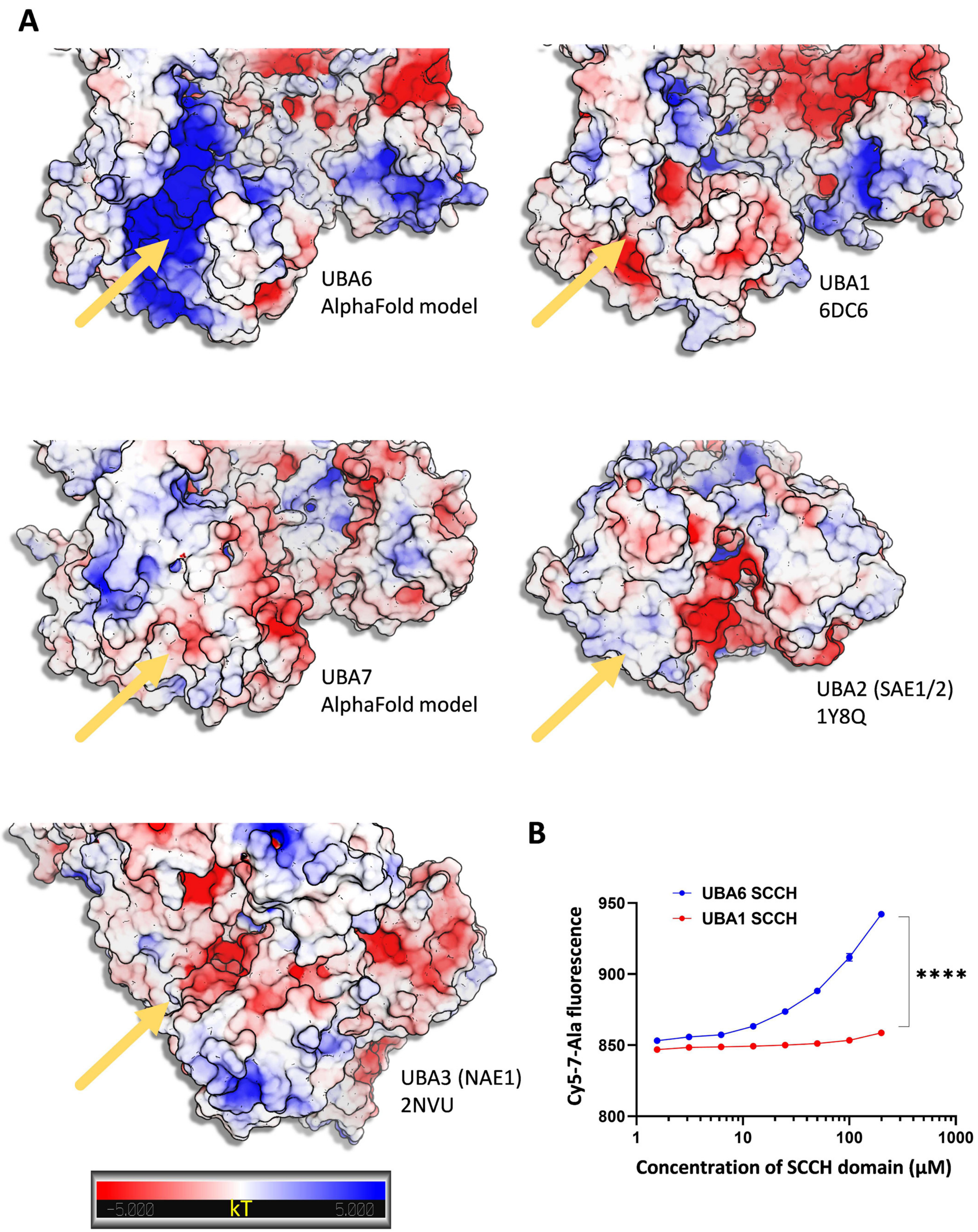
A
UBA6
AlphaFold model
UBA1
6DC6
UBA7
AlphaFold model
UBA2 (SAE1/2)
1Y8Q
UBA3 (NAE1)
2NVU
-5.000
kT
5.000
B
UBA6 SCCH
UBA1 SCCH
Cy5-7-Ala fluorescence
950
900
850
800
1
10
100
1000
****
Concentration of SCCH domain (μM)

 © The Author(s)

◀ **Figure EV2. Biophysical analysis of the interaction between a polyalanine peptide and the SCCH domains of the canonical E1 ubiquitin-like activating enzymes.**

(**A**) AlphaFold models of UBA6 and UBA7 and the crystal structures of UBA1, UBA2 and UBA3 are shown. The structures were aligned and electrostatic potential was calculated as described in the methods. The yellow arrows indicate the location of the groove within the SCCH domains. UBA6, UBA1 and UBA7 form an extended lobe within the SCCH, which is missing in UBA2 and UBA3. The groove in UBA7 is covered and do not exist in UBA2 and UBA3. The grooves in UBA1 and UBA6 are highly similar in terms of structure but present significantly different electro potential surfaces. The gradient from negative (red) to positive (blue) charge is shown. The figure was prepared by PyMol. (**B**) Microscale thermophoresis interaction analysis of cy5-7Ala-peptide against UBA6 or UBA1 SCCH domain. The dose-response curve of cy5-7Ala-peptide titrated against increasing concentrations of the SCCH domain is presented. Results are mean ± s.e.m. $n = 4$ biological replicates, Two-way ANOVA, Sidak's test. ****$P < 0.0001$.

© The Author(s)

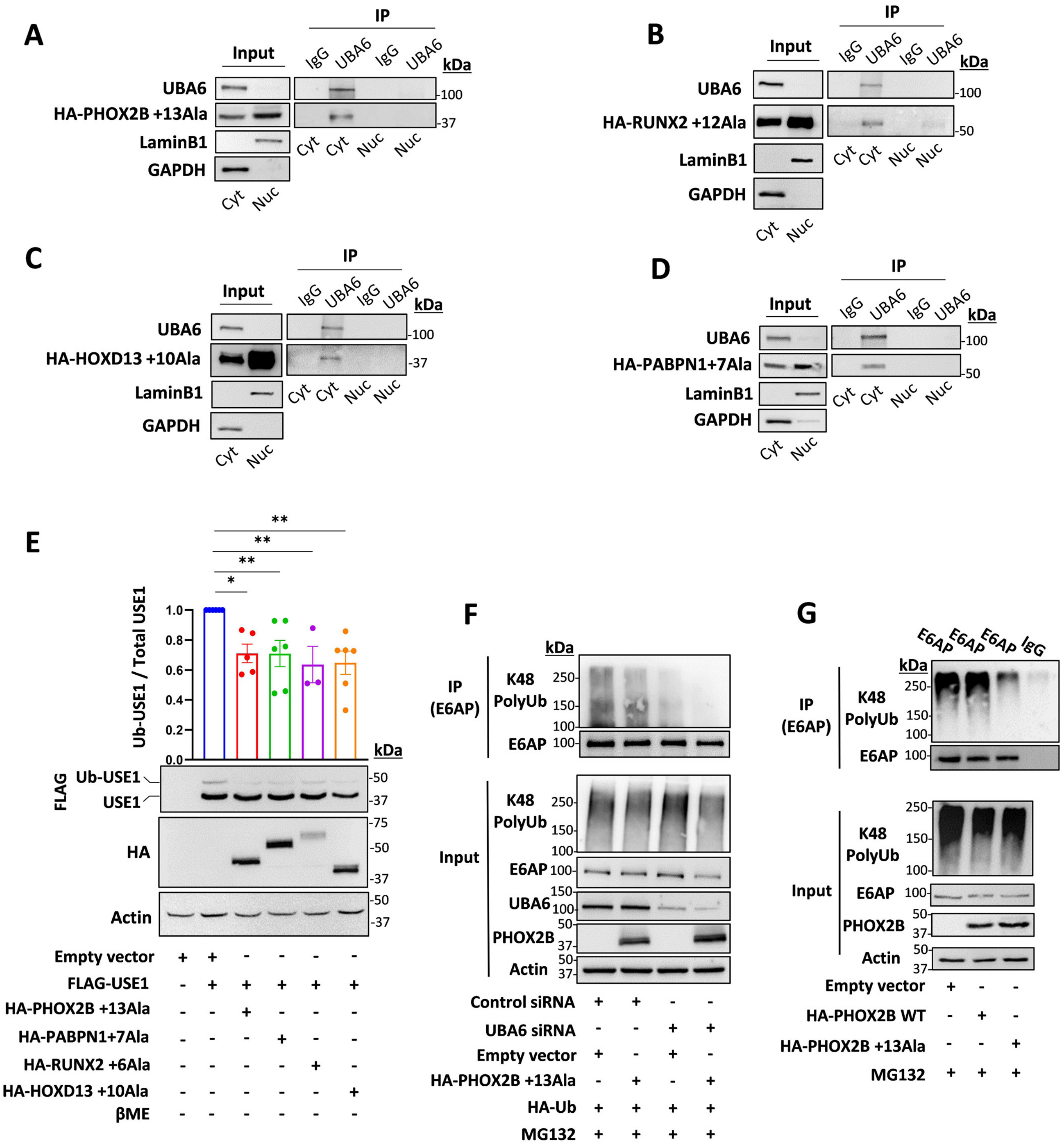
A
Input
IP
IgG UBA6 IgG UBA6
kDa
UBA6
HA-PHOX2B +13Ala
LaminB1
GAPDH
100
37
Cyt Nuc
Cyt Cyt Nuc Nuc
B
Input
IP
IgG UBA6 IgG UBA6
kDa
UBA6
HA-RUNX2 +12Ala
LaminB1
GAPDH
100
50
Cyt Cyt Nuc Nuc
Cyt Nuc
C
Input
IP
IgG UBA6 IgG UBA6
kDa
UBA6
HA-HOXD13 +10Ala
LaminB1
GAPDH
100
37
Cyt Cyt Nuc Nuc
Cyt Nuc
D
Input
IP
IgG UBA6 IgG UBA6
kDa
UBA6
HA-PABPN1+7Ala
LaminB1
GAPDH
100
50
Cyt Cyt Nuc Nuc
Cyt Nuc
E
Ub-USE1 / Total USE1
1.0
0.8
0.6
0.4
0.2
0.0
*
**
**
**
kDa
FLAG
Ub-USE1
USE1
HA
Actin
50
37
75
50
37
50
37
Empty vector + + - - - -
FLAG-USE1 - + + + + +
HA-PHOX2B +13Ala - - + - - -
HA-PABPN1+7Ala - - - + - -
HA-RUNX2 +6Ala - - - - + -
HA-HOXD13 +10Ala - - - - - +
βME - - - - - -
F
kDa
IP (E6AP)
K48 PolyUb
250
150
100
E6AP 100
Input
K48 PolyUb
250
150
100
E6AP 100
UBA6 150 100
PHOX2B 50 37
Actin 50 37
Control siRNA + + - -
UBA6 siRNA - - + +
Empty vector + - + -
HA-PHOX2B +13Ala - + - +
HA-Ub + + + +
MG132 + + + +
G
E6AP E6AP E6AP IgG
kDa
IP (E6AP)
K48 PolyUb
250
150
100
E6AP 100
Input
K48 PolyUb
250
150
100
E6AP 100
PHOX2B 50 37
Actin 50 37
Empty vector + - -
HA-PHOX2B WT - + -
HA-PHOX2B +13Ala - - +
MG132 + + +

 © The Author(s)

Figure EV3. Cytoplasmic polyalanine-expanded disease proteins interact with UBA6 and decrease USE1 ubiquitin loading and E6AP polyubiquitination.

(**A–D**) HEK293T cells were transfected with the indicated constructs: (**A**) HA-mutant PHOX2B (+13Ala). (**B**) HA-mutant RUNX2 (+12Ala). (**C**) HA-mutant HOXD13 (+10Ala). (**D**) HA-mutant PABPN1 (+ 7 Ala). Endogenous UBA6 was immunoprecipitated from the nuclear fraction (Nuc, LaminB1 enriched) or the cytoplasmic (Cyt, GAPDH enriched) fraction (unrelated IgG was used as a control). The immunocomplexes were analyzed with anti-HA antibodies. (**E**) HEK293T cells were transfected with constructs expressing different polyalanine-expanded disease proteins (mutant PHOX2B, mutant RUNX2, mutant HOXD13, and mutant PABPN1) together with FLAG-USE1. Cell lysates were incubated without β mercaptoethanol and analyzed for ubiquitin loading. Results are mean ± s.e.m. normalized to control (empty vector, no disease protein). Paired 2-tailed t test. $n = 3$–6 biological replicates. (**F**) Control and UBA6-depleted HEK293T cells were transfected with HA-Ub, mutant PHOX2B or empty vector and incubated for the last 6 h with the proteasome inhibitor MG132 (10 μM). Endogenous E6AP was immunoprecipitated from cell lysates for ubiquitination analysis. (**G**) HEK293T cells were transfected with WT PHOX2B, mutant PHOX2B or empty vector and incubated for the last 6 h with the proteasome inhibitor MG132 (10 μM). Endogenous E6AP was immunoprecipitated from cell lysates for ubiquitination analysis (unrelated IgG was used as a control). ns non-significant, *$P < 0.05$, **$P < 0.01$.

© The Author(s)

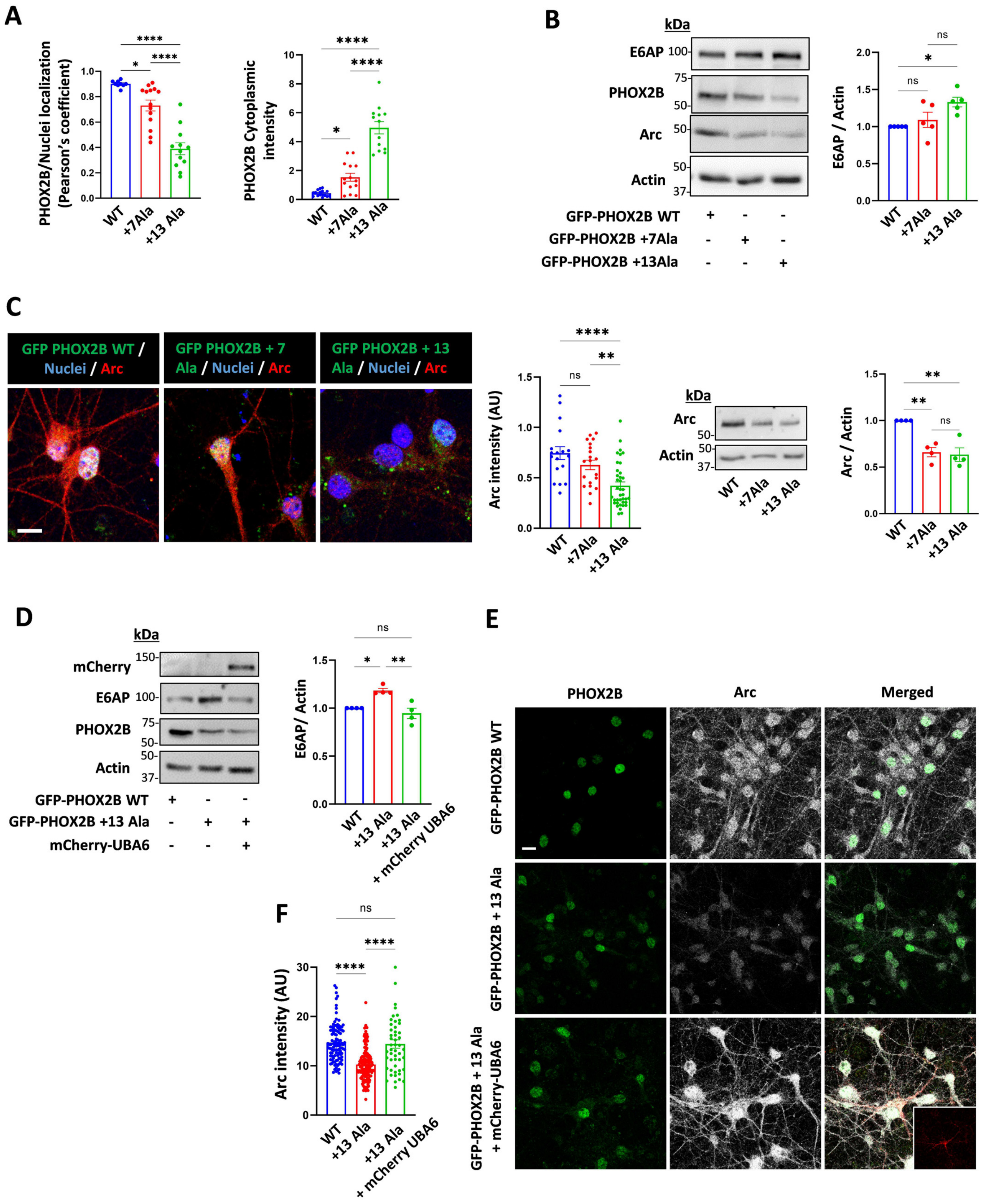
A
PHOX2B/Nuclei localization (Pearson's coefficient)
PHOX2B Cytoplasmic intensity
WT
+7Ala
+13 Ala
B
kDa
E6AP
PHOX2B
Arc
Actin
GFP-PHOX2B WT
GFP-PHOX2B +7Ala
GFP-PHOX2B +13Ala
E6AP / Actin
C
GFP PHOX2B WT / Nuclei / Arc
GFP PHOX2B + 7 Ala / Nuclei / Arc
GFP PHOX2B + 13 Ala / Nuclei / Arc
Arc intensity (AU)
Arc / Actin
D
mCherry
E6AP
PHOX2B
Actin
GFP-PHOX2B +13 Ala
mCherry-UBA6
E6AP/ Actin
+13 Ala + mCherry UBA6
E
PHOX2B
Arc
Merged
GFP-PHOX2B WT
GFP-PHOX2B + 13 Ala
GFP-PHOX2B + 13 Ala + mCherry-UBA6
F
Arc intensity (AU)

 © The Author(s)

Figure EV4. UBA6 overexpression affects E6AP and Arc levels in mutant PHOX2B transduced neurons.

(**A–C**) Mouse primary cortical neurons were transduced with lentiviral vectors expressing GFP-tagged WT PHOX2B or mutant PHOX2B (+7Ala or +13Ala). (**A**) Quantification of the association of GFP-PHOX2B with the nucleus (Pearson's coefficient) is presented as well as GFP-PHOX2B cytoplasmic intensity, related to Fig. 4C. (**B**) Analysis of E6AP and Arc levels in the WT and mutant PHOX2B-expressing neurons ($n = 5$ and $n = 4$ biological replicates, respectively). (**C**) Quantification of Arc intensity in the GFP-PHOX2B expressing neurons is presented (image scale bar 10 μm) as well as a blot showing Arc protein levels. Results represent the average values from neurons in different imaged fields. $n = 20$, $n = 30$ and $n = 100$ neurons analyzed for WT PHOX2B, mutant PHOX2B +7Ala and +13Ala, respectively. (**D–F**) The neurons were transduced with lentiviral vectors expressing GFP-WT PHOX2B, GFP-mutant PHOX2B (+13Ala) with or without lentiviruses encoding for mCherry-UBA6. (**D**), E6AP levels were analyzed in cell lysates. Results are normalized to WT PHOX2B. $n = 4$ biological replicates. (**E**, **F**) Quantification of Arc intensity in cycloheximide-treated GFP-PHOX2B expressing neurons that were positive or negative to mCherry. $n = 110$, $n = 150$ and $n = 50$ neuronal cell bodies analyzed for WT PHOX2B, mutant PHOX2B and mutant PHOX2B + UBA6, respectively. Inset shows mCherry signal. Scale bar 10 μm. AU arbitrary units. Data information: Data points in (**A–D**, **F**) represent mean ± s.e.m. *P* values were calculated by one-way ANOVA Tukey's test (**A–D**, **F**). *$P < 0.05$, **$P < 0.01$, ****$P < 0.0001$, ns non-significant.

© The Author(s)

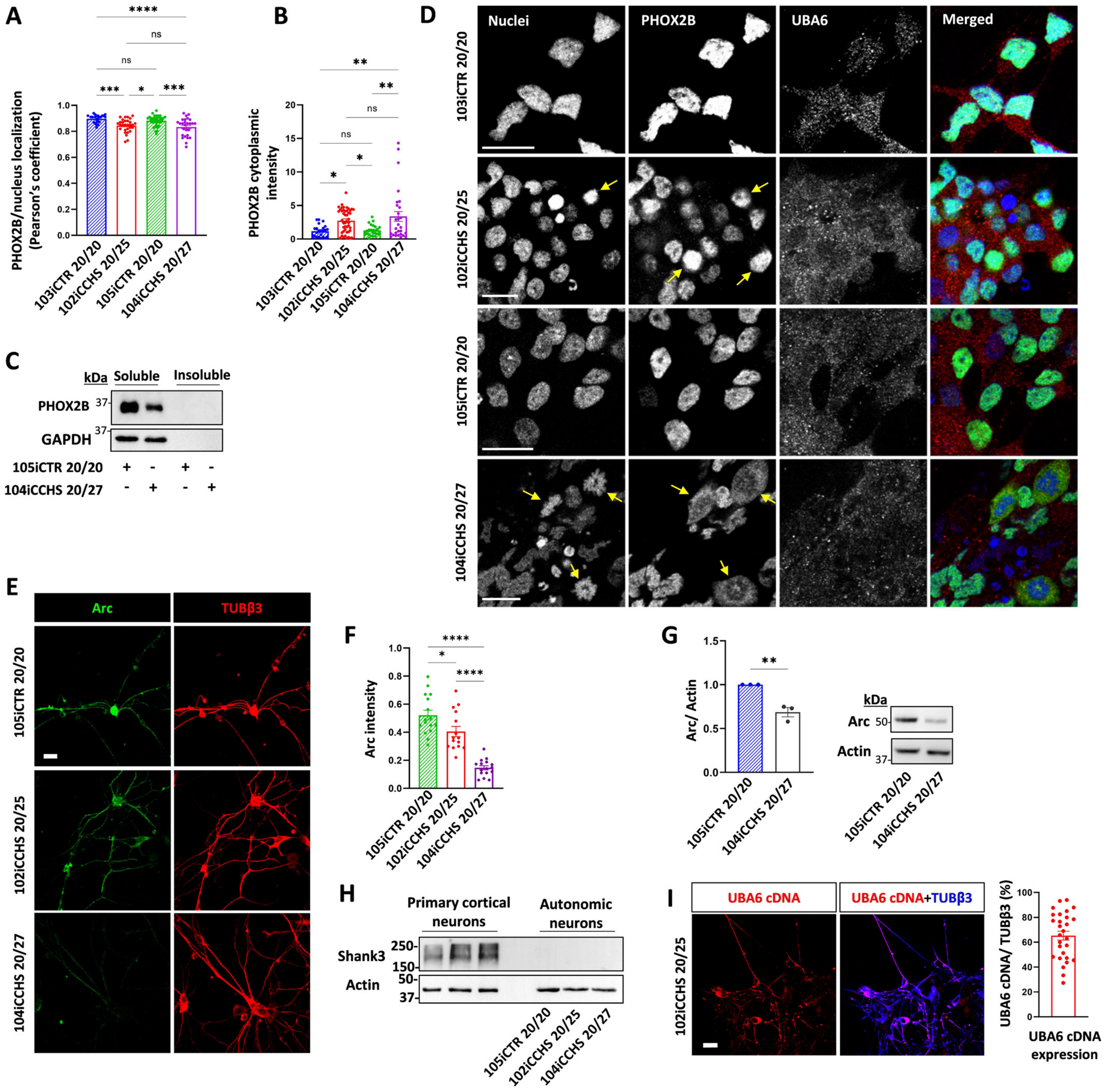
A
PHOX2B/nucleus localization (Pearson's coefficient)
103iCTR 20/20
102iCCHS 20/25
105iCTR 20/20
104iCCHS 20/27
B
PHOX2B cytoplasmic intensity
C
kDa
Soluble
Insoluble
PHOX2B
GAPDH
105iCTR 20/20
104iCCHS 20/27
D
Nuclei
PHOX2B
UBA6
Merged
103iCTR 20/20
102iCCHS 20/25
105iCTR 20/20
104iCCHS 20/27
E
Arc
TUBβ3
105iCTR 20/20
102iCCHS 20/25
104iCCHS 20/27
F
Arc intensity
G
Arc/ Actin
kDa
Arc
Actin
H
Primary cortical neurons
Autonomic neurons
Shank3
Actin
I
UBA6 cDNA
UBA6 cDNA+TUBβ3
102iCCHS 20/25
UBA6 cDNA/ TUBβ3 (%)
UBA6 cDNA expression

 © The Author(s)

Figure EV5. Patient-derived autonomic neurons showing cytoplasmic mislocalized soluble PHOX2B, and decreased Arc levels.

(**A**, **B**) Quantification of the association of endogenous PHOX2B with the nucleus (Pearson's coefficient) in autonomic neurons from control and CCHS patients. Quantification is shown also for PHOX2B cytoplasmic intensity. Total number of neurons analyzed was 160 for 103iCTR 20/20, 350 for 102iCCHS 20/25, 380 for 105iCTR 20/20 and 550 for 104iCCHS 20/27. The results represent additional analysis from the same neurons analyzed in Fig. 5B. (**C**) Autonomic neurons from control and CCHS patients were analyzed for the levels of PHOX2B in the soluble and sarkosyl-insoluble fractions. (**D**) iPSC-derived human autonomic neurons from control and CCHS patients were labeled by nuclear staining (colored blue, abnormal nuclear morphology marked with arrows), and for endogenous PHOX2B (colored green) and endogenous UBA6 (colored red). Images indicate events of severe cytoplasmic mislocalization of PHOX2B (marked with arrows). Scale bar 10 µm. (**E**, **F**) Immunostaining of Arc (colored green) and TUBβ3 (colored red) in autonomic neurons from control and CCHS patients. Scale bar 20µm. For quantification, Arc intensity was normalized to TUBβ3 in different image fields. Number of neurons analyzed 105iCTR 20/20 $n = 105$, 102iCCHS 20/25 $n = 220$, 104iCCHS 20/27 $n = 166$. (**G**) Analysis of Arc levels in the autonomic neurons from control and CCHS patient-derived neurons. Results are normalized to control line. $n = 3$ biological replicates. (**H**) Analysis of shank3 levels in mouse primary cortical neurons and in autonomic neurons from control and CCHS patients. (**I**) Quantification of the abundance of UBA6 cDNA in CCHS patient-derived neurons transduced with mCherry tagged-UBA6 lentiviral vectors. Representative images are presented for mCherry (colored red) and TUBβ3 staining (colored blue) in the patient neurons (scale bar 20 µm). For quantification, the percentage of mCherry coverage from the TUBβ3 staining was calculated in different image fields ($n = 80$ neurons). Data information: Data points in (**A**, **B**, **F**, **G**, **I**) represent mean ± s.e.m. P values were calculated by one-way ANOVA Tukey's test (**A**, **B**, **F**) or paired 2-tailed t test (**G**). $^{*}P < 0.05$, $^{**}P < 0.01$, $^{***}P < 0.001$ $^{****}P < 0.0001$, ns non-significant.

© The Author(s)
